# Assisting Decision-Making on Age of Neutering for 35 Breeds of Dogs: Associated Joint Disorders, Cancers, and Urinary Incontinence

**DOI:** 10.3389/fvets.2020.00388

**Published:** 2020-07-07

**Authors:** Benjamin L. Hart, Lynette A. Hart, Abigail P. Thigpen, Neil H. Willits

**Affiliations:** ^1^Department of Anatomy, Physiology and Cell Biology, School of Veterinary Medicine, University of California, Davis, Davis, CA, United States; ^2^Department of Population Health and Reproduction, School of Veterinary Medicine, University of California, Davis, Davis, CA, United States; ^3^Department of Statistics, University of California, Davis, Davis, CA, United States

**Keywords:** elbow dysplasia, hip dysplasia, cranial cruciate tear, lymphoma, mast cell tumor, hemangiosarcoma, osteosarcoma

## Abstract

Neutering (including spaying) of male and female dogs in the first year after birth has become routine in the U.S. and much of Europe, but recent research reveals that for some dog breeds, neutering may be associated with increased risks of debilitating joint disorders and some cancers, complicating pet owners' decisions on neutering. The joint disorders include hip dysplasia, cranial cruciate ligament tear or rupture, and elbow dysplasia. The cancers include lymphoma, mast cell tumor, hemangiosarcoma, and osteosarcoma. In previous studies on the Golden Retriever, Labrador Retriever and German Shepherd Dog, neutering before a year of age was associated with increased risks of one or more joint disorders, 2–4 times that of intact dogs. The increase was particularly seen with dogs neutered by 6 months of age. In female Golden Retrievers, there was an increase in one or more of the cancers followed to about 2–4 times that of intact females with neutering at any age. The goal of the present study was to expand and use the same data collection and analyses to cover an additional 29 breeds, plus three varieties of Poodles. There were major breed differences in vulnerability to neutering, both with regard to joint disorders and cancers. In most cases, the caregiver can choose the age of neutering without increasing the risks of these joint disorders or cancers. Small-dog breeds seemed to have no increased risks of joint disorders associated with neutering, and in only two small breeds (Boston Terrier and Shih Tzu) was there a significant increase in cancers. To assist pet owners and veterinarians in deciding on the age of neutering a specific dog, guidelines that avoid increasing the risks of a dog acquiring these joint disorders or cancers are laid out for neutering ages on a breed-by-breed and sex basis.

## Introduction

In the U.S. and much of Europe, the practice of neutering male and spaying female dogs (herein both referred to as neutering) has become routine (1) and is increasingly being performed at, or before, 6 months of age. At the same time, several investigations have revealed that joint disorders and some cancers may increase in association with neutering of males and/or females. For example, in studies that did not focus on specific breeds or ages of neutering, one found that hip dysplasia and cranial cruciate ligament tears or ruptures were significantly more likely in neutered than intact males and females (2). Another study found that neutering was associated with a 3-fold increase in excessive tibial plateau angle (3), which is a risk factor for development of cranial cruciate ligament tears or rupture. Neutering is reported to be a risk factor for canine intervertebral disc herniation in Dachshunds (4). Certain cancers are also known to be more likely in neutered than intact dogs. The occurrence of lymphoma was found to be higher in spayed than intact females (5), as was the occurrence of mast cell tumors (6) and hemangiosarcoma (7). A study of over 40,000 dogs utilizing the Veterinary Medical Database found that neutered males and females were more likely to die of cancer than intact dogs (8). A recent finding was that the absence of estrogen from spaying females was associated with accelerated brain aging (9). Another recent report from the Golden Retriever Lifetime Project is that neutering at <6 months increases the risk of cranial cruciate ligament injury (10). Most of the studies cited above offer no useful clinical information or guidelines with regard to the various diseases that may occur in association with neutering in a specific breed.

In an attempt to address the absence of breed-specific information on joint disorders and cancers associated with neutering, we undertook a project focusing on various specific breeds using data collection and analyses with our extensive veterinary hospital database where the same diagnostic criteria could be applied to all breeds. We started with popular breeds well-represented in the database, initially with the Golden Retriever (11, 12), Labrador Retriever (12) and German Shepherd Dog (13). The joint disorders examined included cranial cruciate ligament tears or rupture (CCL), hip dysplasia (HD) and elbow dysplasia (ED). The cancers examined, which previous studies found could be affected by neutering, were lymphoma/lymphosarcoma (LSA), hemangiosarcoma (HSA), mast cell tumors (MCT), and osteosarcoma (OSA).

In the Labrador Retrievers, Golden Retrievers, and German Shepherd Dogs, there was an increase in the incidence of one or more of the joint disorders with neutering in the first year in males and females to 2–4 times >3–5% incidence in intact dogs. In female Golden Retrievers, neutering at any age was associated with an occurrence of one or more of the cancers followed to 2–4 times higher than the 5 percent incidence in intact females. But in male Golden Retrievers, and in male and female Labrador Retrievers and German Shepherd Dogs, there was no evident increase in cancers above that of the dogs left intact. Preliminary analyses from some small-dog breeds revealed no apparent increased risks of joint disorders with neutering. Thus, the research that had been undertaken revealed a wide range of breed-specific differences in disease vulnerability to neutering.

The purpose of this study was to analyze, in a variety of additional breeds, the increased risks, if any, of the above specified joint disorders and cancers associated with neutering male and female dogs at various ages, so as to increase the information available to pet owners and veterinarians for consideration when making decisions regarding neutering specific dogs. We added 29 new breeds to the study, separating three varieties of Poodles, for a total of 32 breed groups (referred to as breeds); this made a total of 35 breeds with the Goldens, Labs and German Shepherds included. The goal was to use the same veterinary hospital database and diagnostic criteria for the diseases as was used with the published studies on the retrievers and German Shepherds so as to allow for direct comparisons among various breeds. The primary purpose was to offer readers some evidence-based information on breed-specific differences with vulnerability to neutering, including suggested guidelines for neutering ages to avoid increasing long-term health risks of neutering, if any. A secondary, unforeseen, purpose was to document breed-specific differences in the increases in some cancers associated with removal of gonadal hormones, as an area for possible research on genetic aspects of cancer occurrence.

## Methods

### Ethics Statement

Hospital records of the Veterinary Medical Teaching Hospital (VMTH) provided the retrospective dataset used. In conformity with the campus policy, faculty of the University of California-Davis, School of Veterinary Medicine, are allowed use of the record system for research purposes. No animal care and use committee approval was required, and strict confidentiality of the owners and their dogs was maintained.

### Subjects Breed Categories

In addition to the Golden Retriever, Labrador Retriever, and German Shepherd Dog, the other breeds chosen for this project included those most frequently occurring in the database and those chosen to obtain a sampling of giant breeds or small-dog breeds. The final list of 35 (including three varieties of Poodle) represented in the present study are, alphabetically, the: Australian Cattle Dog, Australian Shepherd, Beagle, Bernese Mountain Dog, Border Collie, Boston Terrier, Boxer, Bulldog, Cavalier King Charles Spaniel, Chihuahua, Cocker Spaniel, Collie, Corgi (Pembroke and Cardigan combined), Dachshund, Doberman Pinscher, English Springer Spaniel, German Shepherd Dog, Golden Retriever, Great Dane, Irish Wolfhound, Jack Russell Terrier, Labrador Retriever, Maltese, Miniature Schnauzer, Pomeranian, Poodle-Miniature, Poodle-Standard, Poodle-Toy, Pug, Rottweiler, Saint Bernard, Shetland Sheepdog, Shih Tzu, West Highland White Terrier, and Yorkshire Terrier.

### Study Parameters

The present study examined the occurrence in both sexes of the joint disorders: HD, CCL and ED. Also examined in both sexes were the cancers LSA, HSA, MCT, and OSA, because these had been shown in some multi-breed studies to be increased in risks with neutering. In addition, mammary cancer (MC), pyometra (PYO), and urinary incontinence (UI) were examined in female dogs. Of interest was the possible association of early neutering and the occurrence of intervertebral disc disorders (IDD) in the Corgi and Dachshund, two breeds known to be at risk for these diseases. All of the above diseases were examined with regard to dogs neutered in one of the age periods of: <6 mo., 6–11 mo., 1 year (12 to <24 mo.) or 2–8 years, or left intact. The diseases were tracked until the dogs were last seen at the hospital, or through 11 years of age, if seen past their 12th birthday.

Mammary cancer is a late occurring cancer with the median age of diagnosis being 10.1 years in one study (14). Tracking cancers through 11 years of age would be presumably sufficient to catch most cases of MC if the case record had information extending to that age. However, most case records did not extend to that age. As an additional point of comparison, percentages of MC occurrence were looked at in just females tracked through 8 years of age or beyond, including diagnosed MC cases beyond the 12th birthday cut-off, which was the cut-off used for all other data.

### Data Collection and Presentation

The computerized hospital record system of the VMTH provided the dataset. The hospital, with currently over 50,000 cases admitted per year, is a secondary and tertiary facility as well as being a primary care facility. The statistical evaluations, with standardized diagnostic criteria applied to various diseases and taking into account sex and different ages of neutering, required a large database with a computerized record system. The study focused on proportional differences in disease occurrences between the neuter age groups and intact dogs of the same breed and sex.

The study period represented 15 years of data for most breeds. The inclusion criteria were date of birth, age at neutering (if neutered), and age of diagnosis or onset of clinical signs for diseases of interest. As mentioned, age at neutering was designated as <6 mo., 6–11 mo., 1 year (12 to <24 mo.), and 2–8 years (2 to <9 years). The term “early neutering” is sometimes used below to refer to neutering in the first year, combining cases for both the <6 mo. and 6–11 mo. neuter periods. For MC, PYO, and UI, only females were examined. While UI does occur in males, it is predominantly an issue in females.

For all neutered dogs that developed a disease of interest, records were examined to confirm that the dog was neutered prior to the diagnosis or signs of the disease. If the dog developed signs of the disease prior to neutering, the dog was considered intact for analysis of that disease. However, for any disease that occurred after neutering, the dog was considered neutered for analysis of that disease. For any disease of interest that occurred before 12 months of age, the dog was removed from that disease analysis, but included in analyses of other diseases. Therefore, the number of cases for various diseases varied in the analyses for different disease occurrences.

The age at neutering was sometimes not included in the hospital records, so telephone calls to the referring veterinarians were made to obtain the neutering dates or ages. Nonetheless, there were many neutered dogs where age at neutering was not available from the VMTH records or the referring veterinarian, so these dogs were excluded from the study. Of course, this was not an issue with the sample of intact dogs, so there were proportionately more intact cases in the final dataset for each breed than would be expected in the general population. However, the proportion of dogs with a disease, whether intact or neutered, was not affected by the overrepresentation of intact dogs in the database.

The criteria for disease diagnoses were the same as in previous studies on the retrievers and German Shepherd Dog (11–13). A dog was considered as having a disease of interest if the diagnosis was made at the VMTH, or by a referring veterinarian and later confirmed at the VMTH. For joint disorders (HD, ED, and/or CCL), dogs typically presented with signs of lameness, difficulty in moving, and/or joint pain. The diagnosis was confirmed by orthopedic examination, radiographic evidence, and/or surgery. In Dachshunds and Corgis, where intervertebral disc disorders (IDD) is a concern, the diagnosis included herniation, rupture, extrusion, protrusion, fracture, compression, stenosis, or spinal cord injury. For cancers (LSA, HSA, MCT, OSA, MC), the diagnosis was based on the presence of a tissue mass, lumps on the skin or enlarged lymph nodes, and confirmed by chemical panels, appropriate blood cell analyses, imaging, histopathology, and/or cytology. PYO was confirmed by ultrasonic evidence and/or post-surgically after removal of the uterus. UI was confirmed by clinical signs of abnormally frequent urination, urinalyses and exclusion of urinary tract infection and/or other disease. If a diagnosis was listed in the record as “suspected” based on some clinical signs but not confirmed, the case was excluded from the analysis for that specific disease, but the dog was included in other disease analyses.

Although body condition scores have been reported to be a factor in the occurrence of joint disorders (3, 15), our previous studies on the retrievers and German Shepherd Dog found no significant relationship when body condition scores were compared between dogs with and without a joint disorder. Therefore, in the current paper the body condition score is not reported for each breed.

### Statistical Analyses

Survival analysis was used to test for differences with respect to the hazard of a disease in the neutered and intact groups, while adjusting for the differences in time at risk for a disease. The groups were initially compared using a Kaplan Meier life table analysis. *Post-hoc* comparisons among the subgroups were based on least squares means of the hazard within each subgroup. For comparisons where the Kaplan Meier test showed significance at the *p* <0.05 level, both the log-rank and Wilcoxon tests were used for further analyses. Because joint disorders are expected to be seen at a similar risk throughout a dog's lifespan, regardless of age, the log-rank test was used initially for the joint disorders. If the log-rank test did not show significance but the Wilcoxon test did for joint disorders, the Wilcoxon test result was reported with significance level and an asterisk. The reverse rule of thumb was used with cancers where the first test examined was the Wilcoxon test, since the risk of cancer is expected to be higher in older dogs. If the Wilcoxon test did not show significance but the log-rank test did for cancers, the log-rank test result was reported with significance level and an asterisk. For all statistical tests, the two-tailed statistical level of significance was set at *p* <0.05 and reported as either *p* < .05 or *p* <0.01. Each breed was analyzed separately, and there were no statistical comparisons between breeds. However, the overall findings with each breed allow for some general comparisons.

### Data Presentation

For each breed represented on a separate page in Appendix 1, the numbers of intact and neutered males and females are given. In the tables, the percentage of dogs with each of the diseases and the percentage having at least one of the joint disorders and at least one of the cancers (except MC) was calculated for intact males and intact females as well as those neutered at various age ranges. Statistical analyses compared the occurrences of joint disorders and cancers between each neuter period and intact dogs. If the comparison was significant at either the *p* <0.05 or *p* <0.01 level, the data were bolded and the *p*-value was given. The detailed datasets are available online (Figshare, doi: 10.6084/m9.figshare.7231010). Three breeds for which findings have been previously published (Golden Retriever, Labrador Retriever, German Shepherd Dog) are included to present an overall picture in the same Appendix 1. The data for these three breeds were expanded through 11 years of age, to provide continuity among breeds and diseases.

For each breed, a short paragraph summarizes the main findings on joint disorders (HD, CCL, ED), cancers (LSA, HSA, MCT, OSA) for both males and females, and MC, PYO and UI for females. For Dachshunds and Corgis, the occurrence of IDD is listed for both sexes. Survival analyses were not done on IDD occurrence because the condition represented so many different disease diagnoses. Also included in the breed summary information is a suggested guideline for neutering age for males and females to avoid increasing the risks of a disease under consideration. When there was no noticeable occurrence of an increase in joint disorders or cancers with neutering, the guideline statement was made that those wishing to neuter should decide on the appropriate age (or briefly stated as choice in Table 1). When neutering at <6 months was associated with an increased disease risk but no increased risk was evident with neutering beyond 6 months, the default recommended guideline was neutering beyond, 6 months.

**Table 1:** Suggested Guidelines by Breed for Age of Neutering.

	**Suggested guidelines for age of neutering: 35 breeds**									
	**Males**					**Females**				
Australian Cattle Dog		✓						✓		
Australian Shepherd		✓					✓			
Beagle				✓			✓			
Bernese Mt. Dog					✓		✓			
Border Collie				✓					✓	
Boston Terrier				✓			✓			
Boxer					✓					✓
Bulldog		✓					✓			
Cavalier King Charles Spaniel		✓					✓			
Chihuahua		✓					✓			
Cocker Spaniel			✓							✓
Collie		✓							✓	
Corgi			✓				✓			
Dachshund		✓					✓			
Doberman Pinscher	✓									✓
English Springer Spaniel		✓							✓	
German Shepherd					✓					✓
Golden Retriever				✓		✓				
Great Dane		✓					✓			
Irish Wolfhound					✓		✓			
Jack Russell Terrier		✓					✓			
Labrador Retriever			✓						✓	
Maltese		✓					✓			
Miniature Schnauzer		✓					✓			
Pomeranian		✓					✓			
Poodle (Toy)		✓					✓			
Poodle (Miniature)				✓			✓			
Poodle (Standard)					✓		✓			
Pug		✓					✓			
Rottweiler				✓				✓		
Saint Bernard		✓						✓		
Shetland Sheepdog		✓								✓
Shih Tzu		✓								✓
West Highland White Terrier		✓					✓			
Yorkshire Terrier		✓					✓			

*Summary of spaying and neutering guidelines based on findings regarding increased risk of joint disorders and cancers. The term “choice” means there was no increased risk for any age*.

## Results

The breed-by-breed findings are presented in four different formats. One format, seen in this section below, is a short paragraph for each breed. The occurrence of the joint disorders and the cancers followed is reported for the intact and neutered dogs, and the increase in the two disease types over that of the intact dogs, if significant, is reported. Other findings are also mentioned if appropriate, such as IDD occurrence in Dachshunds and Corgis. A second format, represented in Table 1, is a very brief summary of spaying and neutering guidelines based on findings regarding joint disorders and cancers for each breed, allowing the reader to quickly scroll through the various breeds. In the third format, the data-based findings, with statistical notations for each breed, are reported in Appendix 1. In the fourth format, the raw data allowing the reader to perform their own calculations, if desirable, is available in Figshare.

The mean age of last entry was calculated for intact and neutered males and females for each breed and presented in Appendix 2. Across all breeds the mean age of last entry in the record for neutered males was 5.5 years (range 3.71–6.54), for neutered females 5.7 years (range 4.21–6.97), for intact males 4.9 (range 4.15–7.11), and intact females 4.7 (range 3.41–6.32). Upon perusal of the data, it is evident that the mean age of data entry for intact dogs was younger than that of neutered dogs, especially for females, where there is disparity of almost 1 year. To address the issue of whether the lower age of last entry for intact dogs could have resulted in a lower rate of disease occurrence in intact dogs in either joint disorders or cancers, we examined data of dogs where the last entry was at 8 years or beyond. We looked at three breeds with the largest databases (Golden Retrievers, Labrador Retrievers, and German Shepherd Dogs) and where there were significant differences in disease diagnoses between early neutered and intact dogs. Using these parameters, the occurrences of joint disorders in Golden Retrievers for those neutered at ≤ 6mo. vs. intact, in males, there was a 6-fold difference (18% vs. 3%) and in females 3-fold (25 vs. 8%). For male Labrador Retrievers, the figures were 22 vs. 8% and in females 33 vs. 10%. For male German Shepherd Dogs, the figures were 33 vs. 2% and for females, 29 vs. 9%. For cancers in female Goldens, the figures were 26 vs. 14%. The incidence figures, although not sufficient for meaningful statistical analyses, are consistent with the larger database where all ages are included. Thus, while the age of the last visit is a limitation for analyses on late-occurring cancers and joint disorders, the examples chosen for dogs seen at the age of 8 years or beyond are consistent with the overall results presented here; these results appear to represent what would be seen in the general situation.

## General Findings

Looking at the occurrences of these joint disorders and cancers, it is clear that most breeds are unaffected for these diseases by age of neutering. Vulnerability to joint disorders associated with neutering is generally related to body size. Small-dog breeds – Boston Terrier, Cavalier King Charles Spaniel, Chihuahua, Corgi, Dachshund, Maltese, Pomeranian, Poodle-Toy, Pug, Shih Tzu, Yorkshire Terrier – do not appear to have an increased risk in joint disorders with neutering compared to the breeds of larger size. However, in the breeds of larger body size there were differences among the breeds with the two giant breeds – Great Danes and Irish Wolfhounds – showing no indication of increase in one or more joint disorders with neutering at any age.

Although the occurrence of MC was tracked, the female mean age at the last hospital visit for all breeds ended short of the reported, late-onset mean age of MC occurrence in intact female dogs. Thus, the low occurrence of MC in intact females (typically under 6 percent) cannot be expected to represent the actual incidence over a female's lifetime. When the percentage of MC was calculated for only those dogs seen through 8 years of age or older (including cases diagnosed past the 12th birthday), the results did not appear appreciably different than the percentages seen using the study age range. However, the number of dogs seen through age 8 or beyond was fairly small, so the analysis results might change with an increased sample size of these older dogs.

The following are brief summaries for each of the breeds along with suggested guidelines for age of neutering. See Appendix 1 for the complete data set, including statistical analyses for each breed.

### Australian Cattle Dog

The study population was 61 intact males, 58 neutered males, 48 intact females, and 70 spayed females for a total of 237 cases. In this sample, 5 percent of intact males and 2 percent of intact females were diagnosed with one or more joint disorders. Neutering males was not associated with any increased risk in joint disorders, but there was an association with spaying females at <6 mo. where the risk of a joint disorder increased to 15 percent (*p* <0.05). The occurrence of cancers was low for males and females left intact (0 and 3 percent, respectively). There were no evident occurrences of the cancers in dogs neutered at various ages. The occurrence of MC in intact females was 6 percent and in those spayed at 2–8 years, 6 percent. For females left intact, 4 percent were reported with PYO. UI was not reported in any of the spayed or intact females. Lacking a noticeable occurrence of increased joint disorders or cancers in neutered males, those wishing to neuter should decide on the appropriate age. In females, the increased risk of a joint disorder with spaying occurred only at the <6 mo. range, so the suggested guideline is spaying at, or beyond, 6 months.

### Australian Shepherd

The study population was 93 intact males, 135 neutered males, 76 intact females, and 136 spayed females for a total of 440 cases. In this sample, 3 percent of intact males and 4 percent of intact females were diagnosed with one or more joint disorders. Neutering males and females was not associated with any evident increased risk in joint disorders. The occurrence of cancers was 9 percent for intact males and, in contrast, only about 1 percent for intact females. Neutering males did not appear to be associated with an overall increased risk of cancers above the rather high level of intact males. However, spaying females at 6–11 mo. and at 2–8 years was associated with a 7–8 percent risk in cancers which may have reached significance with a larger sample size. The occurrence of MC in intact females was zero, but was 8 percent in females spayed at 2–8 years. For females left intact, 5 percent were reported with PYO. UI was reported in just 1 percent of early-spayed females. Lacking a noticeable occurrence of increased joint disorders or cancers in neutered males, those wishing to neuter should decide on the appropriate age. The guideline for females is the same while also maintaining vigilance for the cancers which may be associated with spaying beyond 6 months, or else leaving the female intact and being vigilant for MC.

### Beagle

The study population was 42 intact males, 82 neutered males, 45 intact females and 87 spayed females for a total of 256 cases. Just 2 percent of intact males were diagnosed with one or more joint disorders, but with neutering at 6–11 mo. joint disorders increased 7-fold to 15 percent, which may have reached significance with a larger sample size. None of the females left intact or spayed had a joint disorder. None of the intact males or females was diagnosed with any of the cancers followed. There was no evident increased occurrence of cancers in neutered males and females. There was no occurrence of MC in intact or late-spayed females. There was 1 case of PYO in intact females (2 percent). UI was reported in only 2 percent of early-spayed females.

For males, in light of a possible increase in joint disorders for those neutered at 6–11 mo., the suggested guideline is to delay neutering males until beyond a year of age. Lacking a noticeable occurrence of increased joint disorders or cancers in neutered females, those wishing to neuter should decide on the appropriate age.

### Bernese Mountain Dog

The study population was 59 intact males, 74 neutered males, 37 intact females, and 65 spayed females for a total of 235 cases. The percentage of intact males with at least one joint disorder was 4 percent and for intact females, 11 percent. Neutering males any time prior to 2 years of age was associated with a significant increase in at least one joint disorder to 23–24%, about a 6-fold increase over intact males (*p* <0.01). Spaying females before 6 mo. increased the likelihood of a joint disorder to over 3-fold that of intact females, but this did not reach significance. The occurrence of one or more of the cancers followed was 9 percent for both intact males and intact females. There was no evident increase in cancer risk in males related to neutering, but with females, spaying at <6 mo. was associated with a 2-fold increase above that of intact females. There was no occurrence of MC in females, whether left intact or neutered at any age, and a 5 percent occurrence of PYO in intact females. There was no occurrence of UI in intact or spayed females. Reflecting the increased risk of joint disorders for males, the suggested guideline for neutering males is delaying neutering until well-beyond 2 years. Lacking a significant occurrence of increased joint disorders or cancers in neutered females, those wishing to neuter should decide on the appropriate age.

### Border Collie

The study population was 105 intact males, 85 neutered males, 88 intact females, and 121 spayed females for a total of 399 cases. In this sample 2–3% of intact males and females were diagnosed with one or more joint disorders, and neutering males and females was not associated with any evident increased risk in joint disorders. The occurrence of one or more of the cancers followed in intact males was 2 percent and none for females left intact. For males, there was a significant increased risk in one or more of the cancers to 13 percent with neutering at 6–11 mo. (*p* <0.05), and for females there was a significant increase in the cancers to 11 percent with spaying at 6–11 mo. (*p* <0.01). The occurrence of MC in intact females was just 1 percent, and for PYO, 4 percent. UI was reported in just one spayed female. The suggested guideline for neutering, given the significant risk of cancers, is holding off neutering of both sexes until beyond a year of age.

### Boston Terrier

The study population was 75 intact males, 67 neutered males, 54 intact females, and 96 spayed females for a total of 291 cases. None of the intact or neutered males or females was diagnosed with one or more joint disorders. For cancers, the story is a bit different in that 5 percent of intact males were diagnosed with one or more cancers and 10 percent of males neutered at <6 mo., and 12 percent of males neutered at 6–11mo. had cancers (*p* <0.01, the two neuter periods combined). For females, 2 percent of intact females had one or more of the cancers and with spaying, there was no evident increase of cancers. The occurrence of MC in intact females was 2 percent and for PYO, 7 percent. UI was 2 percent in early-spayed females. In light of the significant increase in cancers in males with neutering through 11 months of age, the suggested guideline for males is delaying neutering to beyond a year of age. Lacking a noticeable occurrence of increased joint disorders or cancers in neutered females, those wishing to neuter should decide on the appropriate age.

### Boxer

The study population was 220 intact males, 203 neutered males, 128 intact females, and 210 spayed females, for a sample size of 761 cases. Males and females left intact had just a 2 percent occurrence of joint disorders, with neutered males and females showing no apparent increase in this measure. The occurrence of one or more of the cancers followed in intact males was 17 percent, and for intact females, 11 percent. Neutering males before 2 years significantly raised the risk of a cancer over that of intact males to 32 percent (*p* <0.01). The same pattern of increase in cancers was seen in spaying females with up to 20 percent of females having one or more of the cancers with spaying done before 2 years, an increase that was not significant, but with an expanded database may have been. There was no occurrence of MC in intact females. PYO was diagnosed in 2 percent of intact females. Just 1 percent of spayed females were diagnosed with UI. Given the risk of increased cancers, the suggested guideline for both sexes is to delay neutering until beyond 2 years of age.

### Bulldog

The study population was 198 intact males, 156 neutered males, 90 intact females, and 114 spayed females for a sample of 558 cases. The occurrence of joint disorders in intact males was 7 percent and 5 percent in intact females. Neutering at <6 mo. raised the incidence to 15 percent for males and to 18 percent for females, which did not reach significance for either. The cancers followed occurred at the 6 to 7 percent level in intact males and females. There were no significant increases above this with neutering males or females. The occurrence of MC in females left intact was 1 percent and 2 percent with spaying at 2–8 years. There was a 2 percent occurrence of PYO in intact females and no UI in early spayed females. Lacking a significant occurrence of increased joint disorders or cancers in neutered males or females, those wishing to neuter should decide on the appropriate age, but some people may wish to be cautious in view of the possible apparent risk in joint disorders.

### Cavalier King Charles Spaniel

The study population was 51 intact males, 72 neutered males, 87 intact females, and 76 spayed females, for a sample size of 286 cases. For males and females left intact, the occurrences of one or more joint disorders were just 4 and 1 percent, respectively, and for both sexes neutering was not associated with any increase in this measure. The occurrences of cancers in intact males were 2 percent and zero for intact females. For both sexes neutering was not associated with any increase in this measure. The occurrence of MC in females left intact was zero. The occurrence of PYO was 2 percent in intact females. There was no occurrence of UI in spayed females. Lacking a noticeable occurrence of increased joint disorders or cancers in neutered males or females, those wishing to neuter should decide on the appropriate age.

### Chihuahua

The study population was 261 intact males, 189 neutered males, 298 intact females, and 289 spayed females for a total sample of 1,037 cases. For both males and females, neither those left intact, nor those neutered at any age had a noteworthy occurrence of a joint disorder. The cancers followed in both intact and neutered males and females were <5 percent with no evident increase with neutering at any age. The occurrence of MC in females left intact was 1 percent, and in females neutered at 2–8 mo., 4 percent. In intact females, PYO was diagnosed in 2 percent. There was no UI diagnosed in any of the spayed females. Lacking a noticeable occurrence of increased joint disorders or cancers with neutering in either sex, those wishing to neuter should decide on the appropriate age.

### Cocker Spaniel

The study population was 71 intact males, 112 neutered males, 61 intact females, and 127 spayed females, for a sample size of 369 cases. The occurrence of at least one joint disorder was seen in 1 to 3 percent of the intact males and females. Neutering males at <6 mo. was associated with a significant increase of this measure to 11 percent (*p* <0.01). Spaying females was not associated with an increase in joint disorders. The occurrence of one or more of the cancers followed was 6 percent in intact males with no increase with neutering. Although there was no occurrence of cancers in intact females, this measure rose significantly to 17 percent in females spayed between 1 and 2 years of age (*p* <0.01), entirely due to MCT. For females left intact, 11 percent were diagnosed with MC and 5 percent with PYO. None of the spayed females developed UI. The suggested guideline for males is neutering beyond 6 months of age. Given the increased cancer risk for females spayed at a year of age, the suggested guideline is delaying spaying until beyond 2 years of age.

### Collie

The study population was 29 intact males, 26 neutered males, 24 intact females, and 37 spayed females, for a sample size of 116 cases. The occurrence of at least one joint disorder was seen in 7 percent of the intact males and in none of the intact females. None of the neutered males or females had a noteworthy occurrence of a joint disorder. The occurrence of one or more of the cancers followed was 11 percent for intact males and none for the intact females. There was no evident increase of cancers in males with neutering, and in females, there was an increase of cancer to 40 percent in those spayed at <6 mo., which may have reached significance with a larger sample size. For females left intact, 4 percent were diagnosed with MC, and 16 percent were diagnosed with PYO. Of females spayed at 6–11 mo., 13 percent had UI. Lacking a noticeable occurrence of increased joint disorders or cancers in neutered males, those wishing to neuter a male should decide on the appropriate age. For females, given the apparent risks of cancers with spaying at <6 mo. and UI with spaying at 6–11 mo., the guideline is to delay spaying until the female is a year old.

### Corgi (Welsh), Pembroke and Cardigan

The study population was 42 intact males, 78 neutered males, 50 intact females, and 70 spayed females, for a total sample size of 240 cases. Although these are two breeds, they vary only a little in size, so these two breeds are combined for statistical analyses and display of data. The occurrence of at least one joint disorder in intact males was 5 percent and for intact females 6 percent. There was no significant increase in this measure in males or females with neutering. This is one of the breeds where intervertebral disc disorders are a concern, and in 3 percent of intact males and 8 percent of intact females, IDD was reported. In males neutered before 6 months, the occurrence of IDD reached 18 percent, and in females there was no increase with neutering. The occurrence of one or more of the cancers followed was 5 percent in intact males and 6 percent in intact females. In neutered males and females, there was no evident increase in cancers. For females left intact, the occurrence of MC was 8 percent, and there was zero occurrence of PYO. There was no diagnosis of UI in spayed females. The suggested guideline for age of neutering for males, given the increase in IDD with neutering at <6 mo., is beyond 6 months. Lacking a noticeable occurrence of increased joint disorders, IDD, or cancers with neutering females, those wishing to neuter a female should decide on the appropriate age.

### Dachshund

The study population was 177 intact males, 170 neutered males, 99 intact females, and 212 spayed females, for a total sample size of 658 cases. Joint disorders were basically absent in males and females, left intact or neutered. This is a breed plagued by intervertebral disc disorders, and in this sample 53 percent of intact males and 38 percent of intact females were diagnosed with a form of IDD. There was no evident increase in this measure with neutering of males or females. The occurrence of the cancers followed was <1% in both intact males and females, with no indication of an increased risk with neutering. For females left intact, the occurrence of MC was 1 percent and for PYO, 4 percent. None of the spayed females developed UI. Lacking a noticeable occurrence of increased joint disorders or cancers in neutered males or females, those wishing to neuter should decide on the appropriate age.

### Doberman Pinscher

The study population was 109 intact males, 91 neutered males, 53 intact females, and 108 spayed females, for a sample size of 358 cases. The percentage of intact males with at least one joint disorder was 2 percent and 0 percent for intact females. There was no evident increase in this measure with neutering males. For females, spaying within 11 months resulted in an increase in joint disorders of 11 percent, which did not reach significance. The occurrence of one or more of the cancers followed for both intact males and intact females was 2 percent. In neutered males at the 1 year and 2–8 year periods, there was a non-significant increase in occurrence of cancers to 6 percent and 13 percent, respectively. For females, there was no noteworthy increase in cancers with spaying at any time. The occurrence of MC in females left intact was 2 percent and 4 percent for those spayed at 2–8 years. There was a 7 percent occurrence of PYO in intact females. UI was a significant risk in females spayed at any age up to 2 years, ranging from 25 percent in the females spayed at <6 mo. (*p* <0.01) to 19 percent for those spayed between 1 and 2 years (*p* <0.05). The suggested guideline, based on fragmentary results, for males is to leave the male intact or neuter before 1 year of age to avoid the possible increased risk of cancers seen in those neutered beyond a year of age. For females, the suggested guideline, also based on limited data, given the risk of UI in early spayed females, and the possible increased risk of a joint disorder, is to consider delaying spaying until beyond 2 years of age.

### English Springer Spaniel

The study population was 52 intact males, 57 neutered males, 37 intact females, and 66 spayed females for a total sample of 212 cases. In males and females left intact, the occurrence of one or more joint disorders was 5 and 8 percent, respectively. Among males and females neutered at various ages, there were no noteworthy increases in joint disorders. The cancers followed occurred in the intact males and females at a 6 percent level, and neutering at any age was not associated with any evident increase in this measure in either sex. In intact females, MC was diagnosed in 6 percent, and for those spayed at 2–8 years, 15 percent. PYO was not reported in any of the intact females. Spaying females at 6–11 mo. was associated with a 13 percent occurrence of UI, which may have reached significance with a larger sample size. Lacking a noticeable occurrence of increased joint disorders or cancers in neutered males, those wishing to neuter should decide on the appropriate age. For females, given the increased risk of UI in those spayed before 1 year, the suggested guideline is to delay spaying until a year of age.

### German Shepherd Dog

The study population was 514 intact males, 272 neutered males, 173 intact females, and 298 spayed females for a total of 1,257 cases. In males and females left intact, the occurrence of one or more joint disorders was 6 and 5 percent, respectively. Neutering males at <6 mo., 6–11 mo. and 1–2 years was associated with increased risks of this measure to 19, 18 and 9 percent, respectively (*p* <0.01). Spaying females at <6 mo. and 6–11 mo. was associated with a 20 and 15 percent level of increased risk (*p* <0.01), and spaying at 1–2 years with a 5 percent risk level (*p* <0.05). The occurrence of one or more of the cancers followed for intact males and females was 3 percent and 2 percent, respectively. Neutering at the various ages was not associated with any appreciable increased risk in cancers followed. The occurrence of MC in intact females was 5 percent and for those spayed at 2–8 years, 6 percent. Of intact females, 3 percent were reported with PYO. UI ranged up to 9 percent for females spayed from <6 mo. through 1 year of age (*p* <0.05–0.01). The suggested guideline for males, given the risks of joint disorders, is delaying neutering until over 2 years of age. For females, with the same joint issues as males plus the risks of UI, the suggested guideline is delaying spaying until over 2 years of age.

### Golden Retriever

The study population was 318 intact males, 365 neutered males, 190 intact females, and 374 spayed females for a total of 1,247 cases. In intact males and females, the level of occurrence of one or more joint disorders was 5 percent and 4 percent, respectively. Neutering males at <6 mo. and at 6–11 mo. was associated with risks of 25 percent and 11 percent, respectively (*p* <0.01). In females, spaying at <6 mo. and at 6–11 mo. was associated with risks of 18 percent and 11 percent (*p* <0.01, when combined). The occurrence of one or more of the cancers followed in intact males was a high 15 percent and for intact females 5 percent. Neutering males at <6 mo. and at 6–11 mo. was associated with increased risks of cancers to 19 and 16 percent, respectively (*p* <0.01). Spaying females at <6 mo. and at 6–11 mo., was associated with increases in cancers to 11 and 17 percent, respectively (*p* <0.05, when combined) and spaying at 1 year and at 2–8 years was associated with increased risks of 14 percent (*p* <0.01, when combined). The occurrence of MC in intact females was 1 percent and for those spayed at 2–8 years, 4 percent. For females left intact, 4 percent were reported with PYO. No cases of UI were reported in females spayed at any age. The suggested guideline for males, based on the increased risks of joint disorders and cancers, is delaying neutering until beyond a year of age. The suggested guideline for females, based on the increased occurrence of cancers at all spaying ages, is leaving the female intact or spaying at one year and remaining vigilant for the cancers.

### Great Dane

The study population was 90 intact males, 103 neutered males, 69 intact females, and 91 spayed females for a total sample of 353 cases. This is a giant breed where one might expect a high risk of joint disorders. However, both intact males and females have low levels of joint disorders, just 1 and 2 percent, respectively. For both males and females, there was no evident increase in this measure with neutering. The occurrence of one or more of the cancers followed in intact males was 6 percent and for intact females, 3 percent. There was no evident increase in this measure of cancers with neutering in either sex. In intact females, MC was diagnosed in just 2 percent and PYO in 6 percent. In early-spayed females, no UI was reported. Lacking a noticeable occurrence of increased joint disorders or cancers in neutered males or females, those wishing to neuter should decide on the appropriate age. However, given the large body size, and physiology of late musculoskeletal development, neutering well-beyond year 1 should be considered.

### Irish Wolfhound

The study population was 30 intact males, 19 neutered males, 21 intact females, and 16 spayed females for a total of 86 cases. Even with the small number of cases, this breed was chosen for analyses because of the large body size: challenging the Great Dane for height, and where one might expect an increased risk of joint disorders. In this sample, 7 percent of intact males and none of the intact females had a joint disorder. No joint disorders were seen in neutered males or females. With the intact males and females, the incidences of one or more cancers were 8 percent and 21 percent, respectively. With neutering males at 1 year, there was an increase in cancer occurrence to 25 percent (*p* <0.05). There was no evident increase in cancers in neutered females above the relatively high level in intact females. There was no occurrence of MC in intact females or those spayed late. For females left intact, 5 percent were reported with PYO. UI was not reported in any of the spayed or intact females. The suggested guidelines for males given the increased occurrence of cancers around at ages 1–2 years, is neutering beyond 2 years. Lacking a noticeable occurrence of increased joint disorders or cancers in neutered females, those wishing to neuter should decide on the appropriate age. However, given the large body size, and physiology of late musculoskeletal development, some may want to consider neutering females well-beyond year 1.

### Jack Russell Terrier

The study population was 92 intact males, 87 neutered males, 84 intact females, and 113 spayed females for a total sample of 376 cases. As in other small dogs, joint disorders were rare; none of the intact males, and just 2 percent of intact females had one or more joint disorders. Neutering was not associated with any increase in this measure in either sex. In intact males, 3 percent, and in intact females none, had one or more of the cancers followed. There was no evident increase in cancer occurrence in either sex with neutering at any age. In females left intact, MC was seen in 1 percent, as was PYO. In those spayed at 2–8 years, MC was diagnosed in 3 percent. UI was not diagnosed in any females. Lacking a noticeable occurrence of increased joint disorders or cancers in neutered males or females, those wishing to neuter should decide on the appropriate age.

### Labrador Retriever

The study population was 714 intact males, 381 neutered males, 400 intact females, and 438 spayed females for a total of 1,933 cases. One or more joint disorders were reported in 6 percent of both intact males and intact females. This measure was significantly increased to 13 percent for males neutered before 6 mo. (*p* <0.01). In females spayed at <6 mo. and 6–11 mo., the risk of a joint disorder was 11–12 percent for each period (*p* <0.01, spay periods combined). The occurrence of cancers followed was 8 percent and 6 percent, respectively, for intact males and females. Neutering at the various ages was not associated with any evident increased risk in the cancers. The occurrence of MC in intact females was 1 percent and for those spayed at 2–8 years, 2 percent. For females left intact, 2 percent were reported with PYO. UI was reported at a low rate (2–3%) in females spayed at various ages though 1 year. Given the significant occurrence of joint disorders in males neutered at <6 mo., the suggested guideline for males is neutering beyond 6 months. For females, given the increased risks of joint disorders with spaying through 11 months of age, the suggested guideline is delaying spaying until beyond a year of age.

### Maltese

The study population was 49 intact males, 72 neutered males, 65 intact females, and 86 spayed females for a total sample of 272 cases. As mentioned in Appendix 1, the Maltese and Chihuahua vie for the smallest breeds and the Great Dane and Irish Wolfhound for the largest, but all four breeds share a low predisposition to joint disorders. For the Maltese in both sexes, there was no occurrence of joint disorders in either those left intact or neutered. Virtually the same picture emerges with cancers, with only one of 64 intact females being diagnosed with a cancer. There was no occurrence of MC in the intact females and only one case among the 19 females spayed at 2–8 years. PYO was seen in none of the intact females. UI did not occur in any of the females.

Lacking a noticeable occurrence of increased joint disorders or cancers in neutered males or females, those wishing to neuter should decide on the appropriate age.

### Miniature Schnauzer

The study population for this small-dog breed was 47 intact males, 63 neutered males, 25 intact females and 96 spayed females for a total sample of 231 cases. There was virtually no occurrence of any joint disorders in males or females either left intact or neutered. The incidence of cancers in intact males was 4 percent and in females, zero percent. There was no indication of cancer increase related to neutering in either sex. There was no occurrence of MC in any of the females left intact or spayed, and a 4 percent occurrence of PYO in intact females. None of the females was diagnosed with UI. Lacking a noticeable occurrence of increased joint disorders or cancers in neutered males or females, those wishing to neuter should decide on the appropriate age.

### Pomeranian

The study population was 84 intact males, 69 neutered males, 65 intact females, and 104 spayed females for a total sample of 322 cases. As with other dogs of small body size, both males and females had no occurrences of joint disorders in either those left intact or neutered. With regard to cancers, for both males and females left intact, the occurrence of cancers was zero, and there was no indication of increased cancer risk related to neutering in either sex. There was just one case of MC in females left intact, and 7 percent with PYO. None of the females was diagnosed with UI. Lacking a noticeable occurrence of increased joint disorders or cancers in neutered males or females, those wishing to neuter should decide on the appropriate age.

### Poodle, Toy

The study population was 49 intact males, 53 neutered males, 58 intact females, and 78 spayed females for a total sample of 238 cases. While the AKC registers all the Poodle varieties as the same breed, the three main varieties are dealt with separately here because of differences in size. In intact males, 4 percent had one or more joint disorders and in intact females there was no occurrence of a joint disorder. In neutered males and females, there was no evident increased risk of a joint disorder. There was a 2 percent occurrence of cancers in intact males and none in intact females. In neutered males and females, there was no noteworthy occurrence of cancers. In intact females, there was only a single case of MC and no case of PYO in intact females and no occurrence of UI in spayed females. Lacking a noticeable occurrence of increased joint disorders or cancers in neutered males or females, those wishing to neuter should decide on the appropriate age.

### Poodle, Miniature

The study population was 41 intact males, 60 neutered males, 30 intact females, and 69 spayed females for a total sample of 199 cases. The AKC registers the Toy, Miniature, and Standard Poodle varieties, all as the same breed. However, because of differences in size, the varieties of Poodles are dealt with separately here. There was no occurrence of a joint disorder in intact males or females. However, in males neutered at 6-11 mo., there was a significant 9 percent occurrence of joint disorders (*p* <0.01), reflecting CCL. In spayed females, there was no occurrence of a joint disorder. In intact males and females, there was a 5 and zero percent occurrence of cancers, respectively. There was no indication of increased cancer occurrence related to neutering in either sex. The only occurrence of MC in females was one female that had been spayed at 2–8 years. Of intact females, 6 percent developed PYO. Just one female spayed at <6 mo. developed UI. The suggested guideline for males, based on the significant occurrence of a joint disorder with neutering at 6-11 mo., is delaying neutering until a year of age. Lacking a noticeable occurrence of increased joint disorders or cancers in neutered females, those wishing to neuter should decide on the appropriate age.

### Poodle, Standard

The study population was 47 intact males, 88 neutered males, 53 intact females, and 87 spayed females for a total sample of 275 cases. The AKC registers the Toy and Miniature, along with the Standard Poodle, as all being Poodles. However, because of differences in size, the varieties of Poodles are dealt with separately here. There was a 2 percent occurrence of joint disorders in both intact males and females. In males neutered at <6 mo., there was a non-significant increase to 8 percent, and in spayed females, there was no occurrence of joint disorders. The occurrences of cancers in intact males and females were 4 and 2 percent, respectively. In males neutered at 1 year of age, the occurrence of one or more cancers rose to a significant 27 percent (*p* <0.01), all due to the increased risk of LSA. In females, there was no significant increase in cancers with spaying. There was a 4 percent occurrence of MC, and a 2 percent occurrence of PYO in the females left intact. Just one female spayed beyond 2 years later developed UI. The suggested guideline for males, based on the occurrence of one or more cancers with neutering at 1 year, is to delay neutering until 2 years of age. Lacking a noticeable occurrence of increased joint disorders or cancers in neutered females, those wishing to neuter should decide on the appropriate age.

### Pug

The study population was 96 intact males, 106 neutered males, 63 intact females, and 118 spayed females for a total sample of 383 cases. In intact males and females, the occurrences of joint disorders were zero and 2 percent, respectively. In neutered males and females, there was no evident increased occurrence of joint disorders. The level of occurrence of one or more cancers in intact males was 6 percent and in intact females, 8 percent. Neutering males and females did not lead to any evident increase in risk of a cancer. There were no cases of MC in females left intact or spayed at any time, and there was a 5 percent occurrence of PYO in the intact females. None of the females was diagnosed with UI. Lacking a noticeable occurrence of increased joint disorders or cancers in neutered males or females, those wishing to neuter should decide on the appropriate age.

### Rottweiler

The study population was 315 intact males, 152 neutered males, 143 intact females, and 239 spayed females for a total sample of 854 cases. Joint disorders are a major concern in this breed with 8 percent of intact males and 16 percent of intact females having one or more joint disorders. In males, neutering at <6 mo. and at 6-11 mo. resulted in 10 percent and 22 percent occurrences (combined *p* <0.05). In females, spaying at <6 mo. resulted in a significant 43 percent occurrence (*p* <0.05), the main joint disorder being CCL. The cancers followed occurred in the intact males and females at 16 and 11 percent, respectively. These relatively high occurrences of cancers in intact males and females were not increased by neutering at any age. Of females left intact or spayed at 2–8 years, 8 and 5 percent were diagnosed with MC, respectively. In intact females, 12 percent were diagnosed with PYO. With regard to UI, 1 percent of intact females had UI, and in females spayed at <6 mo. and 6-11 mo., 4 and 6 percent, respectively had UI. The suggested guideline for males, given the risk of joint disorders for those neutered at 6-11 mo. or earlier, is neutering beyond a year of age. For females, given the increased risk of joint disorders with neutering at <6 mo., the suggested guideline is spaying beyond 6 months.

### Saint Bernard

The study population was 26 intact males, 27 neutered males, 18 intact females, and 23 spayed females for a total sample of 94 cases. This breed was chosen because of the large size. In intact males and females, the occurrences of one or more joint disorders were 8 percent and 6 percent, respectively. While there was no evident increase in joint disorders with neutering males, in females spayed at <6 mo., joint disorders increased to a significant 100 percent (*p* <0.01). The cancers followed occurred in intact males and females at 4 and 11 percent, respectively. With neutering males and females, there were no noteworthy increases in cancers. There was no occurrence of MC in either the intact or spayed females. In intact females, PYO was diagnosed in 15 percent There was no occurrence of UI in spayed females. Lacking a noticeable occurrence of increased joint disorders or cancers in neutered males those wishing to neuter should decide on the appropriate age. The suggested guideline for females given in the increased risk of joint disorders with neutering at <6 mo., is neutering beyond 6 months. However, given the large body size, some may wish to consider neutering well-beyond 1 year of age.

### Shetland Sheepdog

The study population was 31 intact males, 30 neutered males, 20 intact females, and 52 spayed females for a total sample of 133 cases. There were no joint disorders in intact males and just one in the intact females. In neutered males, the only joint disorder was in one of the males neutered at <6 mo. and in females there was no joint disorder associated with spaying. The occurrence of cancers in intact males was 6 percent and in intact females, zero. There were no evident increases in cancers in neutered males or females. There was no occurrence of MC in intact or spayed females and a 14 percent occurrence of PYO in intact females. Spaying at 6-11 mo. resulted in a 6 percent occurrence of UI, but at 1 year a 33 percent occurrence. Lacking a noticeable occurrence of increased joint disorders or cancers in neutered males, those wishing to neuter should decide on the appropriate age. However, to avoid the high level of UI occurrence in females, one could consider spaying females at, or beyond, 2 years.

### Shih Tzu

The study population was 104 intact males, 112 neutered males, 77 intact females, and 139 spayed females for a total sample of 432 cases. In this small-dog breed there were no occurrences of joint disorders in either intact or neutered males and females, revealing virtually no vulnerability in this regard. There was no occurrence of the cancers followed in intact males and females. In neutered males there was no occurrence of cancers. However, in females, the occurrence of cancers for those spayed at 6-11 mo. was 7 percent and at 1 year this measure reached a significant 18 percent (*p* <0.01). MC occurred in 3 percent of intact females. PYO occurred in 5 percent of intact females. UI was not reported in any females. Lacking a noticeable occurrence of increased joint disorders or cancers in neutered males, those wishing to neuter should decide on the appropriate age. The picture is very different for spaying females where the increased risk of cancers started with spaying at 6-11 mo., reaching 18 percent with spaying at year 1. The suggested guideline for females is to delay spaying until the female is 2 years of age. Another possibility is to spay a female a month or two before 6 months to avoid the increased risk of cancers.

### West Highland White Terrier

The study population was 35 intact males, 33 neutered males, 28 intact females, and 46 spayed females for a total sample of 142 cases. Just one intact male had a joint disorder, and other than this, no joint disorders were reported in intact females or in neutered males or females. None of the intact males or females had any of the cancers followed. There were no noteworthy occurrences of the cancers in neutered males or females. There were no occurrences of MC in either intact or neutered females, and a 7 percent occurrence of PYO in intact females. The occurrence of UI was 14 percent for females spayed at <6 mo. and 6 percent at 6-11 mo. Lacking a noticeable occurrence of increased joint disorders or cancers in neutered males or females, those wishing to neuter should decide on the appropriate age. However, for females, one could consider delaying spaying until a year of age to avoid the risk of UI.

### Yorkshire Terrier

The study population was 134 intact males, 178 neutered males, 144 intact females, and 229 spayed females for a total sample of 685 cases. There were no joint disorders reported in intact males, and in intact females, just 1 percent. In neutered males and females there were no noteworthy occurrences of joint disorders. In intact males and intact females, just 1 percent were reported with at least one of the cancers followed. In both neutered males and females, none of the cancer occurrences was noteworthy. In intact females, the occurrence of MC was 1 percent as was the occurrence with spaying at 2–8 years. PYO was reported in 7 percent of intact females. No UI was reported in any of the intact or spayed females. Lacking a noticeable occurrence of increased joint disorders or cancers in neutered males or females, those wishing to neuter should decide on the appropriate age.

## Discussion

Since the reporting from this center of increased risks of joint disorders and some cancers in Golden Retrievers, Labrador Retrievers, and German Shepherd Dogs (11–13), the appropriate age of neutering has become a common point of discussion (16–18). With the evidence-based information on the risks, if any, of joint disorders, cancers, PYO and UI associated with neutering at different ages for males and females of various as dog breeds, dog owners, and their veterinarians, can use this information to select an age for neutering for the long-term health of their companion dogs on a case-by-case basis.

The overall major finding from the present study is that there are breed differences – and sometimes sex differences – with regard to the increased risks of joint disorders and cancers associated with neutering at various ages. For example, with the Boston Terrier, neutering females at the standard 6 month age did not increase the risks of joint disorders or cancers over that of dogs left intact, but with males, neutering before a year of age was associated with a significant increase in cancers. The opposite effect with genders was seen in the Cocker Spaniel where neutering at 6 months was not associated with an increase in joint disorders or cancers in males, but in females there was a significant increase in risk of cancers to 17 percent with neutering before 2 years.

Another important finding that holds across several breeds is that with the small-dog breeds – Cavalier King Charles Spaniel, Chihuahua, Corgi, Dachshund, Maltese, Pomeranian, Poodle-Toy, Pug, Shih Tzu, Yorkshire Terrier –the occurrences of joint disorders were close to zero in both the intact and neutered males and females. In these small-dog breeds, the occurrence of cancers was low in both those kept intact and neutered. Two exceptions were the Boston Terrier and Shih Tzu where there was there a significant increase in cancers with neutering.

As noted in the results section, the mean date of last entry per patient in the hospital record ranged from about 4.5 to 5.5 years, which means the data especially represent rather early-occurring joint disorders and cancers. The perspective taken here is that it is the early occurring joint disorders and cancers that are the most impactful on the human caregivers, both emotionally and financially, as well as their dogs. To just delay neutering by a year or so to lower the risk of a joint disorder or cancer in those breeds where the issue is relevant, is a noteworthy goal, making it worthwhile to discuss appropriate ages to neuter with caregivers who have a new puppy.

A suggested guideline for the use of the data presented here for those who may wish to focus on a breed or two, is to first scroll through Table 1 to peruse the breeds for a brief look at the neutering guidelines for the breeds of interest. The next step could be to refer to summary paragraphs in the Results section, which present the major findings with a suggested guideline for neutering age. Then for a third step, one could turn to Appendix 1 for detailed joint disorder and cancer tabular data as well as data on MC, PYO, and UI. Our intention is to offer readers data-based information to make case-by-case decisions about age of neutering. As is clearly evident in the breed-specific data presented, one cannot make a generalization for all dogs about age of neutering guidelines.

As mentioned, this study involved 35 breeds, counting the three varieties of Poodles as three breeds. Thus, most breeds registered by AKC or other comparable agencies were not covered. The breeds chosen were the most popular, and with the largest dataset in our records, or were included to sample the largest range of breed sizes as was feasible. Hence, some of the largest breeds (e.g., Great Dane, Irish Wolfhound) and smallest breeds (Miniature Schnauzer, West Highland White Terrier) were included despite lower numbers of patient records. While with some of the most popular breeds there were over 1,000 cases in the database, most breeds ended up with 200 to 500 cases which was sufficient for statistical analyses where the impact of neutering was substantial.

A suggestion for those interested in a breed not covered in this study is to find a breed or two closest genetically to the breed of their interest in order to get an estimate of the various disease risks, if any, associated with neutering. However, one needs to bear in mind that even genetically related breeds may vary a great deal. An example is seen when comparing Golden and Labrador Retrievers, using the data from this study, where in the Labrador, there was no increase in cancer risk above that of intact dogs with neutering, but in the female Golden, the risk of a cancer with neutering increased to 2–4 times that of the 5 percent level of intact females. The popular Poodle breed provides another example, where there are three major varieties in size, the Standard, Miniature, and Toy. In the Standard, neutering males at 1 year was associated with a highly significant increase in the risk of a cancer (mainly LSA) to over six times that of intact males, whereas in the Miniature, there was no increase in cancers with neutering but a significant increase in joint disorders (mainly CCL) with neutering at 6-11 mo.

A likely mechanism by which early neutering may lead to a joint disorder is related to disturbance of the closure of the long-bone growth plates by gonadal hormone secretion as the animal approaches maturity (19, 20). We have proposed that neutering much before the closure of growth plates allows the long bones to grow a little longer than normal, and may sufficiently disturb joint alignments in some neutered dogs to lead to a clinically-apparent joint disorder.

Given the frequency with which early neutering is performed in dogs, it seems surprising that osteoporosis has not been examined given that in humans, chronic loss of gonadal hormones is associated with osteoporosis (21). It could be that the wolf ancestor of the dog had one breeding season and that the bone structure of mature dogs was not as affected by seasonal fluctuations of gonadal hormones as with a permanent gonadal hormonal loss in humans.

One of the frequently mentioned advantages of early neutering of female dogs is protection against MC (22). There may be important genetic, breed-line differences in the occurrence of MC that are not portrayed in our database. However, relevant to the discussion of MC is the recent meta-analysis of published studies on neutering females and MC, finding that the evidence linking neutering to a reduced risk of MC is weak (23). In the data gathered in this study, through 11 years of age, the occurrence of MC in females left intact was rarely above 6 percent and frequently 2 percent or less. For those neutered at <6 months, there was, as expected, no occurrence of MC. Obviously with most cases of intact females not followed through 11 years, and with the 12-year cut-off for those that were followed, many occurrences of MC were missed. However, it seems reasonable, that if MC was a common occurrence in intact females that this disease would have been more frequent in the intact females followed. Further, a very late onset of MC would seem less disturbing to pet owners than the much earlier onsets of joint diseases and other cancers.

For males, there is some concern that neutering beyond puberty will increase the likelihood of a problem behavior such as aggression. However, studies show that while neutering males can reduce aggression to people or other dogs in about 25 percent of males, neutering prior to puberty is no more effective in preventing this problem than is neutering in adulthood in resolving the problem (24, 25).

This paper deals primarily with the risks of diseases that are seen within a given breed and sex. Comparisons between breeds are difficult to interpret, in part because of differences in developmental and physiological factors between breeds including those between smaller and larger breeds. In the text we have reported the occurrences of various diseases in percentages but in statistical analyses the actual data are used. When disease incidence is particularly low in one or more neutering subgroups, the ability to detect significant differences will be low, but there still could be differences which may or may not have been evident in the statistical analyses.

There are at least two major limitations to this study. First, relatively few breeds are covered compared to those included in the various breed registries of kennel clubs and canine organizations. This limitation was necessary so as to apply the same diagnostic criteria for diseases covered across all breeds, using the same database, and the necessity of having sufficient cases for analyses. Second, no information is available as to the reasons the owners or others chose to neuter, or not to neuter their dogs. In California, the vast majority of dogs are neutered, and since 2005 it is legally required for dogs to be neutered prior to adoption from an animal shelter or humane society (26); many breeders impose the same requirement.

In conclusion, the data presented should provide to veterinarians and interested puppy caregivers data-based information for the best age for neutering to avoid increasing the risk of joint disorders and some cancers beyond that of leaving the dog intact. Readers can note that an elevated risk for a joint disorder or cancer occurs in relatively few of these breeds. In other words, with most breeds or sexes, neutering can apparently be done without referral to a particular age, at least with regard to the joint disorders or cancers covered in this study. Of course, individual factors must be taken into account. For puppies of mixed breed, another paper that is currently in press provides data-based information dealing with age of neutering and the risk of one or more joint disorders as a function of the dog adult weight category (27). This information can also help inform decisions on age of recommended neuter in purebred dogs where the breed is not covered in our data.

## Data Availability Statement

The datasets presented in this study can be found in online repositories. The names of the repository/repositories and accession number(s) can be found below: (Figshare, doi: 10.6084/m9.figshare.7231010).

## Author Contributions

BH, LH, and AT: conceived and designed study, collected and complied, and analyzed data. NW: statistical analyses. BH, LH, AT, and NW: drafted and edited manuscript. All authors contributed to the article and approved the submitted version.

## Conflict of Interest

The authors declare that the research was conducted in the absence of any commercial or financial relationships that could be construed as a potential conflict of interest.
