# Supplementary material for: Assisting Decision-Making on Age of Neutering for 35 Breeds of Dogs: Associated Joint Disorders, Cancers, and Urinary Incontinence
Source: Front Vet Sci. 2020 Jul 7;7:388. doi: 10.3389/fvets.2020.00388 (PMC7359819; doi:10.3389/fvets.2020.00388)
Supplement: Supplementary file 1 [file Data_Sheet_1.PDF]

Appendix 1. Complete Dataset and Analyses for Each of 35 Breeds

Australian Cattle Dog

The study population was 61 intact males, 58 neutered males, 48 intact females, and 70 spayed females for a total of 237 cases.

Joint disorders: hip dysplasia (HD), cranial cruciate ligament rupture (CCL), and elbow dysplasia (ED). In this sample, 5 percent of intact males and 2 percent of intact females were diagnosed with one or more joint disorders. Neutering males was not associated with any increased risk in joint disorders, but there was an association with spaying females at <6 mo. with the risk of a joint disorder increased to 15 percent ($p<0.05$*).

Cancers: lymphoma (LSA), mast cell tumor (MCT), hemangiosarcoma (HSA), and osteosarcoma (OSA). The occurrences of cancers were low for males and females left intact (0-3 percent).

Mammary Cancer (MC), Pyometra (PYO) and Urinary Incontinence (UI) in Females. The occurrence of MC in intact females was 6 percent and in those spayed at 2-8 years, 6 percent. For females left intact, 4 percent were reported with PYO. UI was not reported in any of the spayed or intact females.

Bottom line. Lacking a noticeable occurrence of increased joint disorders or cancers with neutering males, those wishing to neuter should decide on the appropriate age. In females, the increased risk of a joint disorder with spaying was just at the < 6 mo. range, so the suggested guideline is spaying beyond 6 months.

Joint disorders. For ages 1 through 11 years, for each neuter period. Bold values indicate significance over the intact group. The asterisk (*) indicates when there was significance using the Wilcoxon test, but no significance using the log-rank test.

	HD	CCL	ED	At Least One
Male < 6 months	0/10 (0)	0/9 (0)	0/10 (0)	0/9 (0)
Male 6 – 11 months	0/14 (0)	0/14 (0)	0/12 (0)	0/12 (0)
Male 1 year	0/14 (0)	0/14 (0)	0/14 (0)	0/14 (0)
Male 2 – 8 years	0/17 (0)	0/17 (0)	0/17 (0)	0/17 (0)
Male Intact	0/61 (0)	3/60 (5)	0/59 (0)	3/59 (5.08)
Female < 6 months	1/13 (7.69)	1/13 (7.69)	0/13 (0)	**2/13 (15.38)***
Female 6 – 11 months	0/23 (0)	0/22 (0)	0/23 (0)	0/22 (0)
Female 1 year	0/10 (0)	0/10 (0)	0/10 (0)	0/10 (0)
Female 2 – 8 years	0/20 (0)	0/20 (0)	0/20 (0)	0/20 (0)
Female Intact	0/48 (0)	1/47 (2.13)	0/48 (0)	1/47 (2.13)

Cancers. For ages 1 through 11 years, for each neuter period.

	LSA	MCT	HSA	OSA	At Least One
Male < 6 months	0/10 (0)	0/10 (0)	0/10 (0)	0/10 (0)	0/10 (0)
Male 6 – 11 months	1/14 (7.14)	0/14 (0)	0/14 (0)	0/14 (0)	1/14 (7.14)
Male 1 year	0/13 (0)	0/14 (0)	0/14 (0)	0/14 (0)	0/13 (0)
Male 2 – 8 years	0/16 (0)	0/17 (0)	0/17 (0)	0/17 (0)	0/16 (0)
Male Intact	1/61 (1.64)	1/61 (1.64)	0/61 (0)	0/61 (0)	2/61 (3.28)
Female < 6 months	0/13 (0)	0/13 (0)	0/13 (0)	0/13 (0)	0/13 (0)
Female 6 – 11 months	1/22 (4.55)	0/23 (0)	0/22 (0)	0/23 (0)	1/21 (4.76)
Female 1 year	0/10 (0)	0/10 (0)	0/10 (0)	0/10 (0)	0/10 (0)
Female 2 – 8 years	0/20 (0)	0/20 (0)	0/20 (0)	2/20 (10)	2/20 (10)
Female Intact	0/48 (0)	0/48 (0)	0/48 (0)	0/47 (0)	0/47 (0)

Australian Shepherd
The study population was 93 intact males, 135 neutered males, 76 intact females, and 136 spayed females for a total of 440 cases.

Joint disorders: hip dysplasia (HD), cranial cruciate ligament rupture (CCL), and elbow dysplasia (ED). In this sample, 3 percent of intact males and 4 percent of intact females were diagnosed with one or more joint disorders. Neutering males and females was not associated with any significantly increased risk in joint disorders.

Cancers: lymphoma (LSA), mast cell tumor (MCT), hemangiosarcoma (HSA), and osteosarcoma (OSA). The occurrence of cancers was 9 percent for intact males and, in contrast, only about 1 percent for intact females. Neutering males did not appear to be associated with an overall increased risk of cancers above the level of intact males. Spaying females at 6-11 mo., and at 2-8 years, was associated with a 7-8 percent risk in cancers which may have reached significance with a larger sample size.

Mammary Cancer (MC), Pyometra (PYO) and Urinary Incontinence (UI) in Females. The occurrence of MC in intact females was zero, but was 8 percent in females spayed at 2-8 years. For females left intact, 5 percent were reported with PYO. UI was reported in just 1 percent of early-spayed females.

Bottom line. Lacking a noticeable occurrence of increased joint disorders or cancers with neutering males, those wishing to neuter should decide on the appropriate age. The guideline for females is the same, but maintaining vigilance for the cancers which may be associated with spaying beyond 6 months, or leaving the female intact and being vigilant for MC.

Joint disorders. For ages 1 through 11 years, for each neuter period.

	HD	CCL	ED	At Least One
Male < 6 months	0/28 (0)	1/28 (3.57)	0/29 (0)	1/27 (3.7)
Male 6 – 11 months	0/54 (0)	1/55 (1.82)	1/56 (1.79)	2/53 (3.77)
Male 1 year	0/20 (0)	0/20 (0)	0/19 (0)	0/19 (0)
Male 2 – 8 years	1/23 (4.35)	1/23 (4.35)	1/24 (4.17)	2/23 (8.7)
Male Intact	1/94 (1.06)	1/92 (1.09)	1/92 (1.09)	3/92 (3.26)
Female < 6 months	0/23 (0)	1/23 (4.35)	0/23 (0)	1/23 (4.35)
Female 6 – 11 months	1/55 (1.82)	1/54 (1.85)	0/54 (0)	2/53 (3.77)
Female 1 year	0/15 (0)	1/15 (6.67)	0/15 (0)	1/15 (6.67)
Female 2 – 8 years	0/38 (0)	0/38 (0)	0/38 (0)	0/37 (0)
Female Intact	1/76 (1.32)	1/76 (1.32)	2/77 (2.6)	3/76 (3.95)

Cancers. For ages 1 through 11 years, for each neuter period.

	LSA	MCT	HSA	OSA	At Least One
Male < 6 months	2/28 (7.14)	1/29 (3.45)	0/29 (0)	0/29 (0)	2/28 (7.14)
Male 6 – 11 months	1/56 (1.79)	0/55 (0)	0/56 (0)	1/56 (1.79)	2/55 (3.64)
Male 1 year	0/20 (0)	0/20 (0)	0/20 (0)	0/20 (0)	0/20 (0)
Male 2 – 8 years	1/21 (4.76)	0/24 (0)	0/23 (0)	1/24 (4.17)	2/21 (9.52)
Male Intact	2/90 (2.22)	1/93 (1.08)	6/91 (6.59)	0/93 (0)	8/88 (9.09)
Female < 6 months	0/23 (0)	0/23 (0)	0/21 (0)	0/22 (0)	0/20 (0)
Female 6 – 11 months	2/54 (3.7)	0/55 (0)	2/55 (3.64)	0/55 (0)	4/54 (7.41)
Female 1 year	0/15 (0)	0/15 (0)	0/15 (0)	0/15 (0)	0/15 (0)
Female 2 – 8 years	0/39 (0)	0/39 (0)	1/38 (2.63)	2/39 (5.13)	3/38 (7.89)
Female Intact	0/76 (0)	0/76 (0)	1/74 (1.35)	0/75 (0)	1/73 (1.37)

Beagle

The study population was 42 intact males, 82 neutered males, 45 intact females, and 87 spayed females for a total of 256 cases.

Joint disorders: hip dysplasia (HD), cranial cruciate ligament rupture (CCL), and elbow dysplasia (ED). Just 2 percent of intact males were diagnosed with one or more joint disorders, but with neutering at 6-11 mo., joint disorders increased to 15 percent, which may have reached significance with a larger sample size. None of the females left intact or spayed, had a joint disorder.

Cancers: lymphoma (LSA), mast cell tumor (MCT), hemangiosarcoma (HSA), and osteosarcoma (OSA). None of the intact males or females was diagnosed with any of the cancers followed. There was no evident increased occurrence of cancers in neutered males and females.

Mammary Cancer (MC), Pyometra (PYO) and Urinary Incontinence (UI) in Females. There was no occurrence of MC in intact or late-spayed females. There was 1 case of PYO in intact females (2 percent). UI was reported in only 2 percent of early-spayed females.

Bottom line. For males, in light of a possible increase in joint disorders for those neutered at 6-11 months, the suggested guideline is to delay neutering males until beyond a year of age. Lacking a noticeable occurrence of increased joint disorders or cancers with neutering females, those wishing to neuter should decide on the appropriate age.

Joint disorders. For ages 1 through 11 years, for each neuter period.

	HD	CCL	ED	At Least One
Male < 6 months	0/27 (0)	0/27 (0)	0/27 (0)	0/27 (0)
Male 6 – 11 months	1/20 (5)	1/20 (5)	1/20 (5)	3/20 (15)
Male 1 year	0/15 (0)	0/15 (0)	0/15 (0)	0/15 (0)
Male 2 – 8 years	0/16 (0)	0/16 (0)	0/16 (0)	0/16 (0)
Male Intact	0/42 (0)	1/41 (2.44)	0/42 (0)	1/41 (2.44)
Female < 6 months	0/15 (0)	0/15 (0)	0/15 (0)	0/15 (0)
Female 6 – 11 months	0/31 (0)	0/31 (0)	0/31 (0)	0/31 (0)
Female 1 year	0/12 (0)	0/12 (0)	0/12 (0)	0/12 (0)
Female 2 – 8 years	0/26 (0)	0/26 (0)	0/26 (0)	0/26 (0)
Female Intact	0/45 (0)	0/45 (0)	0/45 (0)	0/45 (0)

Cancers. For ages 1 through 11 years, for each neuter period.

	LSA	MCT	HSA	OSA	At Least One
Male < 6 months	0/27 (0)	0/27 (0)	1/27 (3.7)	0/27 (0)	1/27 (3.7)
Male 6 – 11 months	0/18 (0)	0/20 (0)	1/20 (5)	0/20 (0)	1/18 (5.56)
Male 1 year	0/15 (0)	0/15 (0)	0/15 (0)	0/15 (0)	0/15 (0)
Male 2 – 8 years	0/16 (0)	1/16 (6.25)	0/16 (0)	0/16 (0)	1/16 (6.25)
Male Intact	0/42 (0)	0/42 (0)	0/42 (0)	0/42 (0)	0/42 (0)
Female < 6 months	0/15 (0)	0/15 (0)	1/15 (6.67)	0/15 (0)	1/15 (6.67)
Female 6 – 11 months	1/29 (3.45)	0/31 (0)	0/31 (0)	0/31 (0)	1/29 (3.45)
Female 1 year	0/12 (0)	0/12 (0)	0/12 (0)	0/12 (0)	0/12 (0)
Female 2 – 8 years	0/25 (0)	0/26 (0)	0/25 (0)	0/26 (0)	0/25 (0)
Female Intact	0/44 (0)	0/45 (0)	0/45 (0)	0/45 (0)	0/44 (0)

Bernese Mountain Dog
The study population was 59 intact males, 74 neutered males, 37 intact females, and 65 spayed females for a total of 235 cases.

Joint disorders: hip dysplasia (HD), cranial cruciate ligament rupture (CCL), and elbow dysplasia (ED). The percentage of intact males with at least one joint disorder was 4 percent and for intact females, 11 percent. Neutering males any time prior to 2 years of age was associated with a significant increase in at least one joint disorder to 23-24 percent, about a 6-fold increase over intact males ($p<0.01$). Spaying females before 6 mo. increased the likelihood of a joint disorder to over 3-fold that of intact females, but this did not reach significance.

Cancers: lymphoma (LSA), mast cell tumor (MCT), hemangiosarcoma (HSA), and osteosarcoma (OSA). The occurrence of one or more of the cancers followed in intact males was 9 percent, and for intact females, 9 percent. There was no evident increase in cancer risk in males related to neutering, but with females, spaying at <6 mo. was associated with a 2-fold increase above that of intact females, which may have reached significance with a larger sample size.

Mammary Cancer (MC), Pyometra (PYO) and Urinary Incontinence (UI) in Females. There was no occurrence of MC in females, whether left intact or neutered at any age, and a 5 percent occurrence of PYO in intact females. There was no occurrence of UI in intact or spayed females.

Bottom line. Reflecting the increased risk of joint disorders for males, the suggested guideline for neutering males is delaying neutering until well beyond 2 years. Lacking a significant occurrence of increased joint disorders or cancers with neutering females, those wishing to neuter should decide on the appropriate age.

Joint disorders. For ages 1 through 11 years, for each neuter period. Bold values indicate significance over the intact group. The dagger (†) indicates significance over the intact group when the early groups (< 6 mo. and 6-11 mo.) are combined.

	HD	CCL	ED	At Least One
Male < 6 months	0/13 (0)	2/15 (13.33)	2/14 (14.29)	**3/13 (23.08)**†
Male 6 – 11 months	2/26 (7.69)	3/28 (10.71)	2/26 (7.69)	**6/25 (24)**†
Male 1 year	0/16 (0)	2/17 (11.76)	1/14 (7.14)	**3/13 (23.08)**
Male 2 – 8 years	0/11 (0)	0/11 (0)	1/11 (9.09)	1/10 (10)
Male Intact	0/54 (0)	0/57 (0)	2/48 (4.17)	2/46 (4.35)
Female < 6 months	0/8 (0)	1/11 (9.09)	2/8 (25)	3/8 (37.5)
Female 6 – 11 months	0/20 (0)	2/22 (9.09)	0/21 (0)	2/19 (10.53)
Female 1 year	0/10 (0)	0/9 (0)	0/9 (0)	0/7 (0)
Female 2 – 8 years	0/20 (0)	0/19 (0)	0/19 (0)	0/19 (0)
Female Intact	2/35 (5.71)	1/37 (2.7)	1/35 (2.86)	4/36 (11.11)

Cancers. For ages 1 through 11 years, for each neuter period.

	LSA	MCT	HSA	OSA	At Least One
Male < 6 months	1/14 (7.14)	0/15 (0)	0/15 (0)	0/15 (0)	1/14 (7.14)
Male 6 – 11 months	2/28 (7.14)	0/30 (0)	0/30 (0)	1/29 (3.45)	3/28 (10.71)
Male 1 year	0/17 (0)	0/17 (0)	0/17 (0)	0/17 (0)	0/17 (0)
Male 2 – 8 years	0/11 (0)	1/12 (8.33)	1/12 (8.33)	0/12 (0)	2/11 (18.18)
Male Intact	2/56 (3.57)	3/58 (5.17)	0/57 (0)	0/59 (0)	5/53 (9.43)
Female < 6 months	1/10 (10)	1/11 (9.09)	0/11 (0)	0/11 (0)	2/11 (18.18)
Female 6 – 11 months	0/23 (0)	1/23 (4.35)	0/23 (0)	0/23 (0)	1/23 (4.35)
Female 1 year	0/11 (0)	0/10 (0)	0/11 (0)	0/11 (0)	0/10 (0)
Female 2 – 8 years	2/19 (10.53)	2/18 (11.11)	0/19 (0)	0/19 (0)	4/16 (25)
Female Intact	0/36 (0)	2/37 (5.41)	0/36 (0)	1/36 (2.78)	3/35 (8.57)

Border Collie

The study population was 105 intact males, 85 neutered males, 88 intact females, and 121 spayed females for a total of 399 cases.

Joint disorders: hip dysplasia (HD), cranial cruciate ligament rupture (CCL), and elbow dysplasia (ED). In this sample 2-3 percent of intact males and females were diagnosed with one or more joint disorders, and neutering males and females was not associated with any evident increased risk in joint disorders.

Cancers: lymphoma (LSA), mast cell tumor (MCT), hemangiosarcoma (HSA), and osteosarcoma (OSA). The occurrence of one or more of the cancers followed in intact males was 2 percent and none for females left intact. For males there was a significant increased risk in one or more of the cancers to 13 percent with neutering at 6-11 mo. ($p<0.05$*). For females there was a significant increase in the cancers to 11 percent with spaying at 6-11 mo. ($p<0.01$).

Mammary Cancer (MC), Pyometra (PYO) and Urinary Incontinence (UI) in Females. The occurrence of MC in intact females was just 1 percent, and for PYO, 4 percent. UI was reported in just one spayed female.

Bottom line. For males and females, given the risks of cancers, the suggested guideline is holding off neutering until at least a year of age.

Joint disorders. For ages 1 through 11 years and for each neuter period.

	HD	CCL	ED	At Least One
Male < 6 months	0/9 (0)	0/8 (0)	0/8 (0)	0/7 (0)
Male 6 – 11 months	0/36 (0)	1/35 (2.86)	0/35 (0)	1/34 (2.94)
Male 1 year	1/9 (11.11)	0/11 (0)	0/11 (0)	1/9 (11.11)
Male 2 – 8 years	0/25 (0)	0/26 (0)	0/26 (0)	0/25 (0)
Male Intact	2/101 (1.98)	1/104 (0.96)	0/105 (0)	3/100 (3)
Female < 6 months	0/24 (0)	0/25 (0)	0/25 (0)	0/24 (0)
Female 6 – 11 months	0/37 (0)	0/37 (0)	0/37 (0)	0/37 (0)
Female 1 year	0/27 (0)	0/27 (0)	0/28 (0)	0/26 (0)
Female 2 – 8 years	0/23 (0)	0/24 (0)	0/25 (0)	0/22 (0)
Female Intact	1/88 (1.14)	1/87 (1.15)	0/88 (0)	2/87 (2.3)

Cancers. For ages 1 through 11 years, for each neuter period. Bold values indicate significance over the intact group. The asterisk (*) indicates when there was significance using the log-rank test, but no significance using the Wilcoxon test. The dagger (†) indicates significance over the intact group when the early groups (< 6 mo. and 6-11 mo.) are combined. The double dagger (‡) indicates significance over the intact group when the early groups (< 6 mo. and 6-11 mo.) are combined when using the log-rank test, but no significance using the Wilcoxon test.

	LSA	MCT	HSA	OSA	At Least One
Male < 6 months	0/8 (0)	0/9 (0)	0/9 (0)	0/9 (0)	0/8 (0)‡
Male 6 – 11 months	2/33 (6.06)	1/36 (2.78)	1/35 (2.86)	0/36 (0)	**4/32 (12.5)*** ‡
Male 1 year	1/11 (9.09)	0/11 (0)	0/11 (0)	0/11 (0)	1/11 (9.09)
Male 2 – 8 years	1/26 (3.85)	0/26 (0)	0/26 (0)	0/25 (0)	1/25 (4)
Male Intact	1/105 (0.95)	0/105 (0)	1/104 (0.96)	0/105 (0)	2/104 (1.92)
Female < 6 months	1/25 (4)	0/25 (0)	0/25 (0)	0/25 (0)	1/25 (4)†
Female 6 – 11 months	2/37 (5.41)	1/37 (2.7)	1/37 (2.7)	1/37 (2.7)	**4/37 (10.81)** †
Female 1 year	1/28 (3.57)	0/28 (0)	0/27 (0)	0/28 (0)	1/27 (3.7)
Female 2 – 8 years	0/23 (0)	1/25 (4)	0/25 (0)	0/25 (0)	1/23 (4.35)
Female Intact	0/84 (0)	0/87 (0)	0/88 (0)	0/88 (0)	0/83 (0)

Boston Terrier

The study population was 75 intact males, 67 neutered males, 54 intact females, and 96 spayed females for a total of 291 cases.

Joint disorders: hip dysplasia (HD), cranial cruciate ligament rupture (CCL), and elbow dysplasia (ED). None of the intact or neutered males, or females, was diagnosed with one or more joint disorders.

Cancers: lymphoma (LSA), mast cell tumor (MCT), hemangiosarcoma (HSA), and osteosarcoma (OSA). For cancers, the story is a bit different in that 5 percent of intact males were diagnosed with one or more cancers followed and 10 percent of males neutered at <6 mo., and 12 percent neutered at 6-11mo. had cancers ($p<0.01$, when the two neuter groups are combined). For females, where 2 percent of intact females had one or more of the cancers followed, there was a non-significant increase of cancers, reflecting one case, with spaying at <6 mo. and 6-11 mo.

Mammary Cancer (MC), Pyometra (PYO) and Urinary Incontinence (UI) in Females. The occurrence of MC in intact females was 2 percent and for PYO 7 percent. UI was 2 percent in early-spayed females.

Bottom Line. For males, in light of the significant increase in cancers with neutering through 11 months of age, the suggested guideline is delaying neutering to beyond a year of age. Lacking a noticeable occurrence of increased joint disorders or cancers with neutering females, those wishing to neuter should decide on the appropriate age.

Joint disorders. For ages 1 through 11 years and for each neuter period.

	HD	CCL	ED	At Least One
Male < 6 months	0/12 (0)	0/11 (0)	0/12 (0)	0/11 (0)
Male 6 – 11 months	0/26 (0)	0/26 (0)	0/26 (0)	0/26 (0)
Male 1 year	0/13 (0)	0/12 (0)	0/13 (0)	0/12 (0)
Male 2 – 8 years	0/15 (0)	0/15 (0)	0/15 (0)	0/15 (0)
Male Intact	0/75 (0)	0/75 (0)	0/75 (0)	0/75 (0)
Female < 6 months	0/18 (0)	0/18 (0)	0/18 (0)	0/18 (0)
Female 6 – 11 months	0/34 (0)	0/34 (0)	0/34 (0)	0/34 (0)
Female 1 year	0/17 (0)	0/17 (0)	0/17 (0)	0/17 (0)
Female 2 – 8 years	0/26 (0)	0/26 (0)	0/26 (0)	0/26 (0)
Female Intact	0/54 (0)	0/54 (0)	0/54 (0)	0/54 (0)

Cancers. For ages 1 through 11 years and for each neuter period. Bold values indicate significance over the intact group. The dagger (†) indicates significance over the intact group when the early groups (< 6 mo. and 6-11 mo.) are combined.

	LSA	MCT	HSA	OSA	At Least One
Male < 6 months	0/10 (0)	1/12 (8.33)	0/12 (0)	0/12 (0)	1/10 (10) †
Male 6 – 11 months	0/24 (0)	1/26 (3.85)	0/25 (0)	2/26 (7.69)	**3/25 (12)** †
Male 1 year	0/13 (0)	0/13 (0)	0/13 (0)	0/13 (0)	0/13 (0)
Male 2 – 8 years	0/15 (0)	1/14 (7.14)	0/15 (0)	0/13 (0)	1/12 (8.33)
Male Intact	1/73 (1.37)	3/76 (3.95)	0/74 (0)	0/75 (0)	4/73 (5.48)
Female < 6 months	0/18 (0)	1/18 (5.56)	0/18 (0)	0/18 (0)	1/18 (5.56)
Female 6 – 11 months	0/32 (0)	1/34 (2.94)	0/34 (0)	0/34 (0)	1/32 (3.13)
Female 1 year	0/17 (0)	0/17 (0)	0/17 (0)	0/17 (0)	0/17 (0)
Female 2 – 8 years	0/25 (0)	0/25 (0)	0/26 (0)	0/25 (0)	0/23 (0)
Female Intact	0/53 (0)	1/54 (1.85)	0/54 (0)	0/54 (0)	1/54 (1.85)

Boxer

The study population was 220 intact males, 203 neutered males, 128 intact females, and 210 spayed females, for a sample size of 761 cases.

Joint disorders: hip dysplasia (HD), cranial cruciate ligament rupture (CCL), and elbow dysplasia (ED). Both males and females appear to have a very low level of one or more joint disorders, with those left intact having just a 2 percent occurrence and neutered males and females showing no apparent increase in this measure.

Cancers: lymphoma (LSA), mast cell tumor (MCT), hemangiosarcoma (HSA), and osteosarcoma (OSA). The occurrence of one or more of the cancers followed in intact males was 17 percent and for intact females, 11 percent. Neutering males at <6 mo. significantly raised the risk of a cancer to 21 percent ($p<0.01$), at 6-11 mo. to 17 percent ($p<0.01$*) and at 1 year to 32 percent ($p<0.01$). The same pattern of increase in cancers was seen in spaying females at various ages, ranging from 14 to 20 percent with spaying done at <6 mo. up to 1 year; these were not significant, but with an expanded database may have been significant.

Mammary Cancer (MC), Pyometra (PYO) and Urinary Incontinence (UI) in Females. There were no occurrences of MC in intact females and no MC in females spayed at any age. PYO was diagnosed in 2 percent of intact females. Just 1-2 percent of spayed females were diagnosed with UI.

Bottom line. Given evidence that neutering before 2 years of age in both sexes increases the occurrence of the cancers followed over that of dogs left intact, the suggested guideline for both sexes is to delay neutering until 2 years of age.

Joint disorders. For ages 1 through 11 years and for each neuter period.

	HD	CCL	ED	At Least One
Male < 6 months	0/30 (0)	1/30 (3.33)	0/30 (0)	1/29 (3.45)
Male 6 – 11 months	0/60 (0)	1/58 (1.72)	0/60 (0)	1/56 (1.79)
Male 1 year	0/39 (0)	1/37 (2.7)	0/39 (0)	1/37 (2.7)
Male 2 – 8 years	0/64 (0)	0/62 (0)	0/65 (0)	0/62 (0)
Male Intact	0/217 (0)	4/219 (1.83)	0/218 (0)	4/214 (1.87)
Female < 6 months	0/48 (0)	1/48 (2.08)	0/50 (0)	1/46 (2.17)
Female 6 – 11 months	0/70 (0)	2/69 (2.9)	0/70 (0)	2/69 (2.9)
Female 1 year	0/29 (0)	0/29 (0)	0/29 (0)	0/29 (0)
Female 2 – 8 years	0/60 (0)	3/58 (5.17)	0/60 (0)	3/58 (5.17)
Female Intact	1/126 (0.79)	3/129 (2.33)	0/127 (0)	3/126 (2.38)

Cancers. For ages 1 through 11 years and for each neuter period. Bold values indicate significance over the intact group. The asterisk (*) indicates when there was significance using the log-rank test, but no significance using the Wilcoxon test. The dagger (†) indicates significance over the intact group when the early groups (< 6 mo. and 6-11 mo.) are combined.

	LSA	MCT	HSA	OSA	At Least One
Male < 6 months	4/30 (13.33)	2/30 (6.67)	0/31 (0)	0/30 (0)	**6/28 (21.43)** †
Male 6 – 11 months	5/56 (8.93)	4/56 (7.14)	0/57 (0)	0/61 (0)	**9/52 (17.31)***†
Male 1 year	5/39 (12.82)	7/37 (18.92)	1/39 (2.56)	0/39 (0)	**12/38 (31.58)**
Male 2 – 8 years	1/61 (1.64)	3/54 (5.56)	2/63 (3.17)	0/65 (0)	5/51 (9.8)
Male Intact	9/210 (4.29)	26/224 (11.61)	2/214 (0.93)	1/218 (0.46)	37/213 (17.37)
Female < 6 months	1/42 (2.38)	6/48 (12.5)	0/47 (0)	0/50 (0)	7/43 (16.28)
Female 6 – 11 months	3/68 (4.41)	5/68 (7.35)	1/67 (1.49)	0/70 (0)	9/64 (14.06)
Female 1 year	3/27 (11.11)	2/27 (7.41)	0/29 (0)	0/28 (0)	5/25 (20)
Female 2 – 8 years	1/57 (1.75)	3/52 (5.77)	0/57 (0)	0/59 (0)	4/46 (8.7)
Female Intact	3/126 (2.38)	11/132 (8.33)	0/125 (0)	0/128 (0)	14/129 (10.85)

Bulldog
The study population was 198 intact males, 156 neutered males, 90 intact females, and 114 spayed females for a sample of 558 cases.

Joint disorders: hip dysplasia (HD), cranial cruciate ligament rupture (CCL), and elbow dysplasia (ED). The occurrence of joint disorders in intact males was 7 percent and 5 percent for females. Neutering at <6 mo. raised the incidence to 15 percent for males and 18 percent for females; this increase did not reach significance in males and females.

Cancers: lymphoma (LSA), mast cell tumor (MCT), hemangiosarcoma (HSA), and osteosarcoma (OSA). The cancers followed occurred at the 6 to 7 percent level in intact males and females. There were no evident increases above this with neutering males or females.

Mammary Cancer (MC), Pyometra (PYO) and Urinary Incontinence (UI) in Females. The occurrence of MC in females left intact was 1 percent and 2 percent with spaying at 2-8 years. There was a 2 percent occurrence of PYO in intact females and no UI in early spayed females.

Bottom line. Lacking a significant occurrence of increased joint disorders or cancers with neutering males or females, those wishing to neuter should decide on the appropriate age.

Joint disorders. For ages 1 through 11 years and for each neuter period.

	HD	CCL	ED	At Least One
Male < 6 months	0/20 (0)	3/21 (14.29)	0/20 (0)	3/20 (15)
Male 6 – 11 months	0/59 (0)	3/57 (5.26)	0/59 (0)	3/55 (5.45)
Male 1 year	1/34 (2.94)	0/34 (0)	0/35 (0)	1/33 (3.03)
Male 2 – 8 years	0/35 (0)	1/33 (3.03)	0/36 (0)	1/33 (3.03)
Male Intact	2/195 (1.03)	12/192 (6.25)	0/198 (0)	13/190 (6.84)
Female < 6 months	1/24 (4.17)	3/22 (13.64)	0/24 (0)	4/22 (18.18)
Female 6 – 11 months	0/28 (0)	1/25 (4)	1/30 (3.33)	2/23 (8.7)
Female 1 year	0/11 (0)	1/10 (10)	0/11 (0)	1/10 (10)
Female 2 – 8 years	0/46 (0)	4/47 (8.51)	0/48 (0)	4/43 (9.3)
Female Intact	1/89 (1.12)	3/88 (3.41)	0/88 (0)	4/87 (4.6)

Cancers. For ages 1 through 11 years and for each neuter period.

	LSA	MCT	HSA	OSA	At Least One
Male < 6 months	0/21 (0)	0/21 (0)	0/19 (0)	0/21 (0)	0/19 (0)
Male 6 – 11 months	6/57 (10.53)	0/58 (0)	0/58 (0)	0/60 (0)	6/55 (10.91)
Male 1 year	0/34 (0)	0/35 (0)	1/33 (3.03)	0/35 (0)	1/33 (3.03)
Male 2 – 8 years	2/34 (5.88)	0/34 (0)	0/36 (0)	0/35 (0)	2/31 (6.45)
Male Intact	9/192 (4.69)	2/197 (1.02)	1/195 (0.51)	0/198 (0)	12/189 (6.35)
Female < 6 months	0/23 (0)	0/24 (0)	0/24 (0)	0/24 (0)	0/23 (0)
Female 6 – 11 months	1/26 (3.85)	0/30 (0)	0/30 (0)	0/30 (0)	1/26 (3.85)
Female 1 year	0/11 (0)	0/11 (0)	0/11 (0)	0/11 (0)	0/11 (0)
Female 2 – 8 years	0/47 (0)	0/44 (0)	1/49 (2.04)	0/48 (0)	1/41 (2.44)
Female Intact	4/88 (4.55)	2/90 (2.22)	0/89 (0)	0/90 (0)	6/87 (6.9)

Cavalier King Charles Spaniel
The study population was 51 intact males, 72 neutered males, 87 intact females, and 76 spayed females, for a sample size of 286 cases.

Joint disorders: hip dysplasia (HD), cranial cruciate ligament rupture (CCL), and elbow dysplasia (ED). For males and females left intact, the occurrences of one or more joint disorders were just 1-4 percent. For males and females neutered at any age, there was no noteworthy increase of joint disorder occurrence, undoubtedly reflecting the small body size.

Cancers: lymphoma (LSA), mast cell tumor (MCT), hemangiosarcoma (HSA), and osteosarcoma (OSA). In intact males, the occurrence of cancers was 2 percent and zero in intact females. For both sexes, neutering was not associated with any increase in this measure.

Mammary Cancer (MC), Pyometra (PYO) and Urinary Incontinence (UI) in Females. The occurrence of MC in females left intact was zero. There was no occurrence of UI in spayed females. PYO was diagnosed in 2 percent of intact females.

Bottom line. Lacking a noticeable occurrence of increased joint disorders or cancers with neutering males or females, those wishing to neuter should decide on the appropriate age.

Joint disorders. For ages 1 through 11 years and for each neuter period.

	HD	CCL	ED	At Least One
Male < 6 months	1/22 (4.55)	0/22 (0)	0/22 (0)	1/22 (4.55)
Male 6 – 11 months	0/31 (0)	1/31 (3.23)	0/30 (0)	1/29 (3.45)
Male 1 year	0/6 (0)	0/6 (0)	0/6 (0)	0/6 (0)
Male 2 – 8 years	0/10 (0)	0/11 (0)	0/11 (0)	0/10 (0)
Male Intact	2/52 (3.85)	0/51 (0)	0/51 (0)	2/52 (3.85)
Female < 6 months	0/17 (0)	0/18 (0)	0/17 (0)	0/17 (0)
Female 6 – 11 months	0/25 (0)	1/25 (4)	0/25 (0)	1/25 (4)
Female 1 year	0/7 (0)	0/8 (0)	0/8 (0)	0/7 (0)
Female 2 – 8 years	0/25 (0)	0/25 (0)	0/25 (0)	0/25 (0)
Female Intact	1/87 (1.15)	0/87 (0)	0/87 (0)	1/87 (1.15)

Cancers. For ages 1 through 11 years and for each neuter period.

	LSA	MCT	HSA	OSA	At Least One
Male < 6 months	0/22 (0)	0/22 (0)	0/22 (0)	0/22 (0)	0/22 (0)
Male 6 – 11 months	1/30 (3.33)	0/32 (0)	0/32 (0)	0/32 (0)	1/30 (3.33)
Male 1 year	0/6 (0)	0/6 (0)	0/6 (0)	0/6 (0)	0/6 (0)
Male 2 – 8 years	0/11 (0)	0/11 (0)	0/11 (0)	0/11 (0)	0/11 (0)
Male Intact	1/49 (2.04)	0/51 (0)	0/51 (0)	0/51 (0)	1/49 (2.04)
Female < 6 months	0/17 (0)	0/18 (0)	0/18 (0)	0/18 (0)	0/17 (0)
Female 6 – 11 months	0/25 (0)	0/25 (0)	0/25 (0)	0/25 (0)	0/25 (0)
Female 1 year	0/8 (0)	0/8 (0)	0/8 (0)	0/8 (0)	0/8 (0)
Female 2 – 8 years	0/25 (0)	0/25 (0)	0/25 (0)	0/25 (0)	0/25 (0)
Female Intact	0/87 (0)	0/87 (0)	0/87 (0)	0/86 (0)	0/86 (0)

Chihuahua

The study population was 261 intact males, 189 neutered males, 298 intact females, and 289 spayed females for a total sample of 1,037 cases.

Joint disorders: hip dysplasia (HD), cranial cruciate ligament rupture (CCL), and elbow dysplasia (ED). Undoubtedly reflecting the small body size, for both males and females, those left intact and neutered at any age had a low level of joint disorder occurrence (less than 3 percent).

Cancers: lymphoma (LSA), mast cell tumor (MCT), hemangiosarcoma (HSA), and osteosarcoma (OSA). The cancers followed occurred at a low level in both intact and neutered males and females (less than 5 percent) with no evident increase from neutering at any age.

Mammary Cancer (MC), Pyometra (PYO) and Urinary Incontinence (UI) in Females. The occurrence of MC in females left intact was 1 percent, and in females neutered at 2-8 mo., 4 percent. In intact females, PYO was diagnosed in 2 percent. There was no UI diagnosed in any of the spayed females.

Bottom line. Lacking a noticeable occurrence of increased joint disorders or cancers with neutering in either sex, those wishing to neuter should decide on the appropriate age.

Joint disorders. For ages 1 through 11 years and for each neuter period.

	HD	CCL	ED	At Least One
Male < 6 months	0/26 (0)	0/26 (0)	0/26 (0)	0/26 (0)
Male 6 – 11 months	0/65 (0)	1/65 (1.54)	0/65 (0)	1/65 (1.54)
Male 1 year	0/37 (0)	1/37 (2.7)	0/37 (0)	1/37 (2.7)
Male 2 – 8 years	0/55 (0)	1/55 (1.82)	0/55 (0)	1/55 (1.82)
Male Intact	0/261 (0)	0/261 (0)	0/261 (0)	0/261 (0)
Female < 6 months	0/24 (0)	0/24 (0)	0/24 (0)	0/24 (0)
Female 6 – 11 months	0/78 (0)	1/78 (1.28)	0/78 (0)	1/78 (1.28)
Female 1 year	0/71 (0)	0/71 (0)	0/71 (0)	0/71 (0)
Female 2 – 8 years	0/106 (0)	0/106 (0)	0/107 (0)	0/105 (0)
Female Intact	1/297 (0.34)	0/298 (0)	0/297 (0)	1/296 (0.34)

Cancers. For ages 1 through 11 years and for each neuter period.

	LSA	MCT	HSA	OSA	At Least One
Male < 6 months	0/26 (0)	0/25 (0)	0/26 (0)	0/26 (0)	0/25 (0)
Male 6 – 11 months	1/63 (1.59)	2/65 (3.08)	0/65 (0)	0/65 (0)	3/63 (4.76)
Male 1 year	0/37 (0)	0/37 (0)	0/37 (0)	0/37 (0)	0/37 (0)
Male 2 – 8 years	0/55 (0)	0/55 (0)	0/55 (0)	0/55 (0)	0/55 (0)
Male Intact	4/261 (1.53)	4/261 (1.53)	0/261 (0)	0/261 (0)	8/261 (3.07)
Female < 6 months	0/24 (0)	0/24 (0)	0/24 (0)	0/24 (0)	0/24 (0)
Female 6 – 11 months	0/77 (0)	2/78 (2.56)	0/78 (0)	0/78 (0)	2/77 (2.6)
Female 1 year	0/71 (0)	0/71 (0)	0/71 (0)	0/71 (0)	0/71 (0)
Female 2 – 8 years	0/106 (0)	1/106 (0.94)	0/107 (0)	1/107 (0.93)	2/105 (1.9)
Female Intact	0/293 (0)	2/299 (0.67)	0/297 (0)	0/298 (0)	2/294 (0.68)

Cocker Spaniel

The study population was 71 intact males, 112 neutered males, 61 intact females, and 127 spayed females, for a sample size of 369 cases.

Joint disorders: hip dysplasia (HD), cranial cruciate ligament rupture (CCL), and elbow dysplasia (ED). Occurrences of at least one joint disorder were seen in 1 to 3 percent of the intact males and females, respectively. Neutering males at <6 mo. was associated with a significant increase of this measure to 11 percent (*p*<0.01). Spaying females was not associated with an increase in joint disorders.

Cancers: lymphoma (LSA), mast cell tumor (MCT), hemangiosarcoma (HSA), and osteosarcoma (OSA). The occurrence of one or more of the cancers followed was 6 percent in intact males and this was not increased in males neutered at any age. Although there were no occurrences of cancers in intact females, this measure rose significantly to 17 percent in females spayed between 1 and 2 years of age (*p*<0.01), entirely due to MCT.

Mammary Cancer (MC), Pyometra (PYO) and Urinary Incontinence (UI) in Females. For females left intact, 11 percent were diagnosed with MC and 5 percent with PYO. None of the spayed females developed UI.

Bottom line. The suggested guideline for males is neutering beyond 6 months of age. This avoids the increased risk of a joint disorder with neutering before 6 months. Given the increased cancer risk for females spayed at a year of age, the suggested guideline is delaying spaying until beyond 2 years of age, or spaying at 6-11 months.

Joint disorders. For ages 1 through 11 years and for each neuter period. Bold values indicate significance over the intact group. The dagger (†) indicates significance over the intact group when the early groups (< 6 mo. and 6-11 mo.) are combined.

	HD	CCL	ED	At Least One
Male < 6 months	0/20 (0)	2/19 (10.53)	0/20 (0)	**2/19 (10.53)** †
Male 6 – 11 months	0/32 (0)	1/32 (3.13)	0/32 (0)	1/32 (3.13) †
Male 1 year	0/20 (0)	0/20 (0)	0/20 (0)	0/20 (0)
Male 2 – 8 years	0/35 (0)	0/35 (0)	0/35 (0)	0/35 (0)
Male Intact	0/71 (0)	1/71 (1.41)	0/70 (0)	1/71 (1.41)
Female < 6 months	0/23 (0)	0/23 (0)	0/23 (0)	0/23 (0)
Female 6 – 11 months	0/36 (0)	0/36 (0)	0/36 (0)	0/36 (0)
Female 1 year	0/12 (0)	0/12 (0)	0/12 (0)	0/12 (0)
Female 2 – 8 years	1/51 (1.96)	0/51 (0)	0/51 (0)	1/51 (1.96)
Female Intact	0/61 (0)	2/61 (3.28)	0/61 (0)	2/61 (3.28)

Cancers. For ages 1 through 11 years and for each neuter period. Bold values indicate significance over the intact group.

	LSA	MCT	HSA	OSA	At Least One
Male < 6 months	1/20 (5)	0/20 (0)	0/20 (0)	0/20 (0)	1/20 (5)
Male 6 – 11 months	0/32 (0)	0/31 (0)	0/32 (0)	0/32 (0)	0/31 (0)
Male 1 year	1/20 (5)	0/20 (0)	0/20 (0)	0/20 (0)	1/20 (5)
Male 2 – 8 years	0/35 (0)	0/35 (0)	0/35 (0)	0/35 (0)	0/35 (0)
Male Intact	3/69 (4.35)	1/70 (1.43)	0/71 (0)	0/70 (0)	4/67 (5.97)
Female < 6 months	0/23 (0)	1/23 (4.35)	0/23 (0)	0/23 (0)	1/23 (4.35)
Female 6 – 11 months	0/36 (0)	0/36 (0)	0/36 (0)	0/36 (0)	0/36 (0)
Female 1 year	0/12 (0)	2/12 (16.67)	0/12 (0)	0/12 (0)	**2/12 (16.67)**
Female 2 – 8 years	0/51 (0)	1/50 (2)	1/51 (1.96)	1/51 (1.96)	3/50 (6)
Female Intact	0/61 (0)	0/61 (0)	0/60 (0)	0/61 (0)	0/60 (0)

Collie
The study population was 29 intact males, 26 neutered males, 24 intact females, and 37 spayed females, for a sample size of 116 cases.

Joint disorders: hip dysplasia (HD), cranial cruciate ligament rupture (CCL), and elbow dysplasia (ED). The occurrence of at least one joint disorder was seen in 7 percent of the intact males, but in none of the neutered males. In females, none of those left intact had a joint disorder, but a joint disorder was seen in one female spayed at 6-11 mo.

Cancers: lymphoma (LSA), mast cell tumor (MCT), hemangiosarcoma (HSA), and osteosarcoma (OSA). Occurrences of one or more of the cancers followed were 11 percent in intact males with no significant occurrences above this level in those neutered. No intact females were diagnosed with a cancer, but a non-significant increase in cancer occurrence was seen in females spayed at <6 mo.

Mammary Cancer (MC), Pyometra (PYO) and Urinary Incontinence (UI) in Females. For females left intact, 4 percent were diagnosed with MC, and 16 percent were diagnosed with PYO. UI was diagnosed in 13 percent of females spayed at 6-11 mo.

Bottom line. Lacking a noticeable occurrence of increased joint disorders or cancers with neutering males, those wishing to neuter a male should decide on the appropriate age. For females, given the apparent risks of cancers with spaying at < 6 mo. and UI with spaying at 6-11 mo., the guideline is to delay spaying until the female is a year old.

Joint disorders. For ages 1 through 11 years and for each neuter period.

	HD	CCL	ED	At Least One
Male < 6 months	0/3 (0)	0/3 (0)	0/3 (0)	0/3 (0)
Male 6 – 11 months	0/12 (0)	0/12 (0)	0/12 (0)	0/12 (0)
Male 1 year	0/3 (0)	0/3 (0)	0/3 (0)	0/3 (0)
Male 2 – 8 years	0/7 (0)	0/7 (0)	0/7 (0)	0/7 (0)
Male Intact	1/29 (3.45)	0/29 (0)	1/29 (3.45)	2/29 (6.9)
Female < 6 months	0/5 (0)	0/4 (0)	0/5 (0)	0/4 (0)
Female 6 – 11 months	0/6 (0)	1/8 (12.5)	0/8 (0)	1/7 (14.29)
Female 1 year	0/6 (0)	0/6 (0)	0/6 (0)	0/6 (0)
Female 2 – 8 years	0/16 (0)	0/16 (0)	0/16 (0)	0/16 (0)
Female Intact	0/24 (0)	0/24 (0)	0/24 (0)	0/24 (0)

Cancers. For ages 1 through 11 years and for each neuter period.

	LSA	MCT	HSA	OSA	At Least One
Male < 6 months	0/3 (0)	0/3 (0)	0/3 (0)	1/3 (33.33)	1/3 (33.33)
Male 6 – 11 months	0/11 (0)	0/12 (0)	0/12 (0)	0/12 (0)	0/11 (0)
Male 1 year	0/3 (0)	0/3 (0)	0/3 (0)	0/3 (0)	0/2 (0)
Male 2 – 8 years	1/7 (14.29)	0/7 (0)	0/7 (0)	0/7 (0)	1/7 (14.29)
Male Intact	2/28 (7.14)	0/29 (0)	0/29 (0)	1/29 (3.45)	3/28 (10.71)
Female < 6 months	1/5 (20)	0/5 (0)	1/5 (20)	0/5 (0)	2/5 (40)
Female 6 – 11 months	0/8 (0)	0/8 (0)	0/8 (0)	0/8 (0)	0/8 (0)
Female 1 year	0/6 (0)	0/6 (0)	0/6 (0)	0/6 (0)	0/6 (0)
Female 2 – 8 years	0/16 (0)	0/16 (0)	0/16 (0)	1/16 (6.25)	1/16 (6.25)
Female Intact	0/22 (0)	0/24 (0)	0/23 (0)	0/24 (0)	0/22 (0)

Corgi (Welsh), Pembroke and Cardigan

The study population was 42 intact males, 78 neutered males, 50 intact females, and 70 spayed females, for a total sample size of 240 cases. There are two breeds making up the Welsh Corgi, that vary a little in size, but because of small numbers, the cases for these two breeds are combined for statistical analyses and display of data.

Joint disorders: hip dysplasia (HD), cranial cruciate ligament rupture (CCL), and elbow dysplasia (ED). The occurrence of at least one joint disorder was 5 percent for males and 6 percent for females. There were non-significant increases in this measure in males and females to 9 percent and 8 percent, respectively, with neutering at <6 mo.

Intervertebral disc disorders (IDD). In intact males and females of this breed, the occurrence of IDD is 3 percent in males and 8 percent in females. The only increase seen was with neutering males before 6 months where the occurrence reached 18 percent.

Cancers: lymphoma (LSA), mast cell tumor (MCT), hemangiosarcoma (HSA), and osteosarcoma (OSA). Occurrences of one or more of the cancers followed were 5 percent in intact males and 6 percent in intact females. In neutered males and females, there was no evident increase in cancers.

Mammary Cancer (MC), Pyometra (PYO) and Urinary Incontinence (UI) in Females. For females left intact, the occurrence of MC was at 8 percent, and there was zero occurrence of PYO. There was no diagnosis of UI in spayed females.

Bottom line. The suggested guideline for males, given the increase in IDD with neutering at < 6 mo., is neutering beyond 6 months. Lacking a noticeable occurrence of increased joint disorders, IDD, or cancers with neutering females, those wishing to neuter a female should decide on the appropriate age.

Joint disorders. For ages 1 through 11 years and for each neuter period.

	HD	CCL	ED	At Least One
Male < 6 months	2/22 (9.09)	0/23 (0)	0/23 (0)	2/22 (9.09)
Male 6 – 11 months	0/30 (0)	0/31 (0)	0/31 (0)	0/30 (0)
Male 1 year	0/5 (0)	0/5 (0)	0/5 (0)	0/5 (0)
Male 2 – 8 years	0/17 (0)	0/17 (0)	0/17 (0)	0/17 (0)
Male Intact	1/42 (2.38)	1/41 (2.44)	0/42 (0)	2/41 (4.88)
Female < 6 months	0/13 (0)	1/12 (8.33)	0/13 (0)	1/12 (8.33)
Female 6 – 11 months	1/24 (4.17)	1/24 (4.17)	0/24 (0)	2/24 (8.33)
Female 1 year	0/6 (0)	0/6 (0)	0/5 (0)	0/5 (0)
Female 2 – 8 years	0/23 (0)	0/23 (0)	0/23 (0)	0/23 (0)
Female Intact	2/50 (4)	1/49 (2.04)	0/50 (0)	3/49 (6.12)

Cancers. For ages 1 through 11 years and for each neuter period.

	LSA	MCT	HSA	OSA	At Least One
Male < 6 months	1/23 (4.35)	0/23 (0)	1/23 (4.35)	0/23 (0)	2/23 (8.7)
Male 6 – 11 months	1/31 (3.23)	0/31 (0)	0/31 (0)	0/31 (0)	1/31 (3.23)
Male 1 year	0/5 (0)	0/5 (0)	0/5 (0)	0/5 (0)	0/5 (0)
Male 2 – 8 years	1/16 (6.25)	0/17 (0)	0/17 (0)	0/17 (0)	1/16 (6.25)
Male Intact	2/42 (4.76)	0/42 (0)	0/42 (0)	0/42 (0)	2/42 (4.76)
Female < 6 months	0/12 (0)	0/13 (0)	0/12 (0)	0/13 (0)	0/11 (0)
Female 6 – 11 months	1/23 (4.35)	0/24 (0)	1/24 (4.17)	0/24 (0)	2/23 (8.7)
Female 1 year	0/6 (0)	0/6 (0)	0/6 (0)	0/6 (0)	0/6 (0)
Female 2 – 8 years	1/23 (4.35)	0/23 (0)	0/23 (0)	0/23 (0)	1/23 (4.35)
Female Intact	3/51 (5.88)	0/50 (0)	0/50 (0)	0/50 (0)	3/51 (5.88)

Dachshund

The study population was 177 intact males, 170 neutered males, 99 intact females, and 212 spayed females, for a total sample size of 658 cases.

Joint disorders: hip dysplasia (HD), cranial cruciate ligament rupture (CCL), and elbow dysplasia (ED). The occurrence of joint disorders was basically absent in males and females, whether left intact or neutered.

Intervertebral disc disorders (IDD). This is a breed plagued by intervertebral disc disorders. In this sample 53 and 38 percent of intact males and females, respectively, were diagnosed with a form of IDD. There was no evident increase in this measure with neutering of males, and for females spayed at <6 mo. and at 6-11 mo., there were occurrences of 61 and 59 percent, respectively.

Cancers: lymphoma (LSA), mast cell tumor (MCT), hemangiosarcoma (HSA), and osteosarcoma (OSA). The occurrence of the cancers followed was less than 1 percent in both intact males and females, with no indication of an increased risk with neutering.

Mammary Cancer (MC), Pyometra (PYO) and Urinary Incontinence (UI) in Females. For females left intact, the occurrence of MC was 1 percent and for PYO, 4 percent. None of the spayed females developed UI.

Bottom line. Lacking a noticeable occurrence of increased joint disorders or cancers with neutering males or females, those wishing to neuter should decide on the appropriate age.

Joint disorders. For ages 1 through 11 years and for each neuter period.

	HD	CCL	ED	At Least One
Male < 6 months	0/38 (0)	0/38 (0)	0/38 (0)	0/38 (0)
Male 6 – 11 months	0/47 (0)	0/47 (0)	0/47 (0)	0/47 (0)
Male 1 year	0/22 (0)	0/22 (0)	0/22 (0)	0/22 (0)
Male 2 – 8 years	0/56 (0)	0/56 (0)	0/56 (0)	0/56 (0)
Male Intact	0/177 (0)	0/177 (0)	0/177 (0)	0/177 (0)
Female < 6 months	0/29 (0)	0/29 (0)	0/29 (0)	0/29 (0)
Female 6 – 11 months	0/90 (0)	0/89 (0)	0/90 (0)	0/89 (0)
Female 1 year	0/28 (0)	0/28 (0)	0/28 (0)	0/28 (0)
Female 2 – 8 years	1/58 (1.72)	0/58 (0)	0/58 (0)	1/58 (1.72)
Female Intact	0/98 (0)	0/99 (0)	0/99 (0)	0/98 (0)

Cancers. For ages 1 through 11 years and for each neuter period.

	LSA	MCT	HSA	OSA	At Least One
Male < 6 months	0/38 (0)	0/38 (0)	0/38 (0)	0/38 (0)	0/38 (0)
Male 6 – 11 months	0/47 (0)	0/47 (0)	1/47 (2.13)	0/47 (0)	1/47 (2.13)
Male 1 year	0/22 (0)	0/22 (0)	0/22 (0)	0/22 (0)	0/22 (0)
Male 2 – 8 years	0/56 (0)	1/56 (1.79)	0/56 (0)	0/56 (0)	1/56 (1.79)
Male Intact	0/176 (0)	1/177 (0.56)	0/177 (0)	0/177 (0)	1/176 (0.57)
Female < 6 months	0/29 (0)	0/29 (0)	1/29 (3.45)	0/29 (0)	1/29 (3.45)
Female 6 – 11 months	0/90 (0)	1/90 (1.11)	0/90 (0)	0/90 (0)	1/90 (1.11)
Female 1 year	0/28 (0)	0/27 (0)	0/28 (0)	0/28 (0)	0/27 (0)
Female 2 – 8 years	0/56 (0)	0/58 (0)	0/58 (0)	0/58 (0)	0/56 (0)
Female Intact	0/97 (0)	0/99 (0)	0/99 (0)	0/99 (0)	0/97 (0)

Doberman Pinscher

The study population was 106 intact males, 91 neutered males, 53 intact females, and 108 spayed females, for a sample size of 358 cases.

Joint disorders: hip dysplasia (HD), cranial cruciate ligament rupture (CCL), and elbow dysplasia (ED). The percentage of intact males with at least one joint disorder was 2 percent. There were no occurrences of this measure at neuter dates up to 2 years, but in the 2-8 year neuter period, there was a non-significant increase in joint disorders to 5 percent. For females, with no joint disorders in intact dogs, spaying within 11 months (<6 mo. and 6-11mo. combined) resulted in a non-significant increase in joint disorders up to 11 percent**.**

Cancers: lymphoma (LSA), mast cell tumor (MCT), hemangiosarcoma (HSA), and osteosarcoma (OSA). The occurrences of one or more of the cancers followed for intact males and intact females were 2 percent. In males neutered at the 1 year and 2-8 year periods, there was a non-significant increase in occurrences of cancers to 6 percent and 13 percent, respectively, which may have been significant with a larger sample size. In females, there was no noteworthy increase in cancers with spaying at any time.

Mammary Cancer (MC), Pyometra (PYO) and Urinary Incontinence (UI) in Females. The occurrences of MC in those females left intact and those spayed at 2-8 years were 4 percent. There was a 7 percent occurrence of PYO in intact females. UI was a significant risk in females spayed at any age up to 2 years, ranging from 25 percent in the females spayed at < 6 mo. ($p < 0.01$) to 19 percent for those spayed between 1 and 2 years ($p < 0.05$).

Bottom line. For males, the suggested guideline, based on limited data, is to leave the male intact, or to neuter before 1 year of age to avoid the possible increased risk of cancers seen in those neutered beyond a year of age. For females, the suggested guideline that again is based on limited data, given the risk of both UI in early-spayed females and possible increase in joint disorders, is to consider delaying spaying until beyond 2 years of age.

Joint disorders. For ages 1 through 11 years and for each neuter period.

	HD	CCL	ED	At Least One
Male < 6 months	0/4 (0)	0/4 (0)	0/4 (0)	0/4 (0)
Male 6 – 11 months	0/21 (0)	0/21 (0)	0/21 (0)	0/21 (0)
Male 1 year	0/18 (0)	0/18 (0)	0/18 (0)	0/18 (0)
Male 2 – 8 years	1/43 (2.33)	1/41 (2.44)	0/43 (0)	2/41 (4.88)
Male Intact	0/106 (0)	2/106 (1.89)	0/105 (0)	2/105 (1.9)
Female < 6 months	0/19 (0)	2/18 (11.11)	0/19 (0)	2/18 (11.11)
Female 6 – 11 months	0/28 (0)	3/28 (10.71)	0/29 (0)	3/27 (11.11)
Female 1 year	0/24 (0)	1/24 (4.17)	0/24 (0)	1/24 (4.17)
Female 2 – 8 years	0/34 (0)	0/33 (0)	0/34 (0)	0/33 (0)
Female Intact	0/53 (0)	0/52 (0)	0/53 (0)	0/52 (0)

Cancers. For ages 1 through 11 years and for each neuter period.

	LSA	MCT	HSA	OSA	At Least One
Male < 6 months	0/4 (0)	0/4 (0)	0/4 (0)	0/4 (0)	0/4 (0)
Male 6 – 11 months	0/19 (0)	0/21 (0)	0/21 (0)	0/20 (0)	0/18 (0)
Male 1 year	0/18 (0)	0/18 (0)	0/18 (0)	1/18 (5.56)	1/18 (5.56)
Male 2 – 8 years	4/42 (9.52)	1/42 (2.38)	0/42 (0)	1/41 (2.44)	5/38 (13.16)
Male Intact	1/105 (0.95)	1/105 (0.95)	0/106 (0)	0/105 (0)	2/103 (1.94)
Female < 6 months	0/17 (0)	0/19 (0)	0/19 (0)	0/16 (0)	0/15 (0)
Female 6 – 11 months	1/27 (3.7)	0/29 (0)	0/29 (0)	1/28 (3.57)	2/26 (7.69)
Female 1 year	0/24 (0)	0/23 (0)	0/24 (0)	0/24 (0)	0/23 (0)
Female 2 – 8 years	0/32 (0)	0/34 (0)	0/31 (0)	1/32 (3.13)	1/29 (3.45)
Female Intact	1/51 (1.96)	0/53 (0)	0/53 (0)	0/53 (0)	1/51 (1.96)

English Springer Spaniel

The study population was 52 intact males, 57 neutered males, 37 intact females, and 66 spayed females for a total sample of 212 cases.

Joint disorders: hip dysplasia (HD), cranial cruciate ligament rupture (CCL), and elbow dysplasia (ED). In males and females left intact, the occurrence of one or more joint disorders was 5 to 8 percent, respectively. Among males and females neutered at various ages, there were no significant increases in joint disorders above the levels seen in intact dogs.

Cancers: lymphoma (LSA), mast cell tumor (MCT), hemangiosarcoma (HSA), and osteosarcoma (OSA). The cancers followed occurred in the intact males and females at a 6 percent level, and neutering at any age was not associated with any significant increase in this measure.

Mammary Cancer (MC), Pyometra (PYO) and Urinary Incontinence (UI) in Females. In intact females, MC was diagnosed in 6 percent. This cancer occurred in 15 percent of females spayed at 2-8 years. PYO was not reported in any of the intact females. Spaying females at 6-11 mo. was associated with an occurrence of UI at the 13 percent level which may have reached significance with a larger sample.

Bottom line. Lacking a noticeable occurrence of increased joint disorders or cancers with neutering males, those wishing to neuter should decide on the appropriate age. For females, given the increased risk of UI in those spayed before 1 year, the suggested guideline is to delay spaying until a year of age.

Joint disorders. For ages 1 through 11 years and for each neuter period.

	HD	CCL	ED	At Least One
Male < 6 months	0/14 (0)	0/14 (0)	1/13 (7.69)	1/13 (7.69)
Male 6 – 11 months	1/14 (7.14)	1/14 (7.14)	0/14 (0)	1/14 (7.14)
Male 1 year	1/10 (10)	0/10 (0)	0/11 (0)	1/10 (10)
Male 2 – 8 years	0/14 (0)	0/15 (0)	0/15 (0)	0/14 (0)
Male Intact	3/52 (5.77)	0/51 (0)	2/52 (3.85)	4/52 (7.69)
Female < 6 months	0/9 (0)	0/9 (0)	0/9 (0)	0/9 (0)
Female 6 – 11 months	0/31 (0)	1/31 (3.23)	0/31 (0)	1/31 (3.23)
Female 1 year	0/7 (0)	0/7 (0)	0/7 (0)	0/7 (0)
Female 2 – 8 years	0/16 (0)	2/16 (12.5)	0/16 (0)	2/16 (12.5)
Female Intact	1/37 (2.7)	1/37 (2.7)	0/37 (0)	2/37 (5.41)

Cancers. For ages 1 through 11 years and for each neuter period.

	LSA	MCT	HSA	OSA	At Least One
Male < 6 months	0/14 (0)	0/14 (0)	0/14 (0)	0/14 (0)	0/14 (0)
Male 6 – 11 months	0/14 (0)	0/14 (0)	0/14 (0)	0/14 (0)	0/14 (0)
Male 1 year	1/11 (9.09)	0/11 (0)	0/11 (0)	0/11 (0)	1/11 (9.09)
Male 2 – 8 years	0/14 (0)	0/15 (0)	0/15 (0)	0/15 (0)	0/14 (0)
Male Intact	3/50 (6)	0/52 (0)	0/51 (0)	0/52 (0)	3/49 (6.12)
Female < 6 months	0/9 (0)	0/9 (0)	0/9 (0)	0/9 (0)	0/9 (0)
Female 6 – 11 months	1/29 (3.45)	0/30 (0)	0/29 (0)	0/31 (0)	1/29 (3.45)
Female 1 year	0/7 (0)	0/7 (0)	0/7 (0)	0/7 (0)	0/7 (0)
Female 2 – 8 years	0/16 (0)	1/16 (6.25)	0/16 (0)	0/16 (0)	1/16 (6.25)
Female Intact	1/37 (2.7)	1/37 (2.7)	0/36 (0)	0/37 (0)	2/36 (5.56)

German Shepherd Dog

The study population was 514 intact males, 272 neutered males, 173 intact females, and 298 spayed females for a total of 1,257 cases.

Joint disorders: hip dysplasia (HD), cranial cruciate ligament rupture (CCL), and elbow dysplasia (ED). In males and females left intact, the occurrences of one or more joint disorders were 6 and 5 percent, respectively. Neutering males at <6 mo., 6-11 mo. and 1-2 years was associated with increased risks of this measure to 19, 18 and 9 percent, respectively ($p<0.01$). Spaying females at <6 mo. and 6-11 mo. was associated with 20 and 15 percent levels of increased risk ($p<0.01$), and spaying at 1-2 years with a 5 percent risk level ($p<0.05$).

Cancers: lymphoma (LSA), mast cell tumor (MCT), hemangiosarcoma (HSA), and osteosarcoma (OSA). The occurrences of one or more of the cancers followed were 3 percent and 2 percent, respectively, for intact males and females. Neutering at the various ages was not associated with any significant increased risk in cancers followed.

Mammary Cancer (MC), Pyometra (PYO) and Urinary Incontinence (UI) in Females. The occurrence of MC in intact females was 5 percent and for those spayed at 2-8 years, 6 percent. For females left intact, 3 percent were reported with PYO. UI ranged up to 9 percent for females spayed from <6 mo. through 1 year of age ($p<0.05 – 0.01$).

Bottom line. For males, given the significant increase in joint disorders with neutering prior to 2 years of age, the suggested guideline is delaying neutering until over 2 years of age. For females, with the same joint issues as males and the risks of UI, the suggested guideline is delaying spaying until over 2 years of age.

Joint disorders. For ages 1 through 11 years and for each neuter period. Bold values indicate significance over the intact group. The dagger (†) indicates significance over the intact group when the early groups (< 6 mo. and 6-11 mo.) are combined.

	HD	CCL	ED	At Least One
Male < 6 months	2/26 (7.69)	3/28 (10.71)	1/27 (3.7)	**5/26 (19.23)** †
Male 6 – 11 months	4/55 (7.27)	5/63 (7.94)	3/58 (5.17)	**10/55 (18.18)** †
Male 1 year	2/56 (3.57)	1/59 (1.69)	2/58 (3.45)	**5/56 (8.93)**
Male 2 – 8 years	2/99 (2.02)	1/107 (0.93)	1/109 (0.92)	3/97 (3.09)
Male Intact	23/496 (4.64)	3/511 (0.59)	8/508 (1.57)	31/491 (6.31)
Female < 6 months	4/47 (8.51)	5/54 (9.26)	0/49 (0)	**9/46 (19.57)** †
Female 6 – 11 months	6/75 (8)	5/80 (6.25)	0/78 (0)	**11/72 (15.28)** †
Female 1 year	1/38 (2.63)	1/39 (2.56)	0/39 (0)	**2/38 (5.26)**
Female 2 – 8 years	4/103 (3.88)	1/110 (0.91)	1/110 (0.91)	6/102 (5.88)
Female Intact	6/171 (3.51)	1/172 (0.58)	1/171 (0.58)	8/168 (4.76)

Cancers. For ages 1 through 11 years and for each neuter period.

	LSA	MCT	HSA	OSA	At Least One
Male < 6 months	0/28 (0)	0/28 (0)	0/28 (0)	0/28 (0)	0/28 (0)
Male 6 – 11 months	3/63 (4.76)	0/63 (0)	0/63 (0)	0/63 (0)	3/63 (4.76)
Male 1 year	0/59 (0)	0/59 (0)	0/59 (0)	0/59 (0)	0/59 (0)
Male 2 – 8 years	1/110 (0.91)	0/108 (0)	1/110 (0.91)	1/110 (0.91)	3/108 (2.78)
Male Intact	8/502 (1.59)	2/512 (0.39)	5/508 (0.98)	2/511 (0.39)	17/499 (3.41)
Female < 6 months	0/52 (0)	0/53 (0)	0/54 (0)	0/54 (0)	0/51 (0)
Female 6 – 11 months	2/81 (2.47)	0/80 (0)	2/82 (2.44)	0/82 (0)	4/80 (5)
Female 1 year	0/38 (0)	0/38 (0)	0/39 (0)	1/39 (2.56)	1/37 (2.7)
Female 2 – 8 years	1/108 (0.93)	1/110 (0.91)	0/108 (0)	1/110 (0.91)	3/105 (2.86)
Female Intact	2/171 (1.17)	0/173 (0)	1/172 (0.58)	0/173 (0)	3/171 (1.75)

Golden Retriever

The study population was 318 intact males, 365 neutered males, 190 intact females, and 374 spayed females for a total of 1,247 cases.

Joint disorders: hip dysplasia (HD), cranial cruciate ligament rupture (CCL), and elbow dysplasia (ED). In intact males and females the levels of occurrence of one or more joint disorders were 5 percent for males and 4 percent for females. Neutering males at <6 mo. and at 6-11 mo. was associated with risks of 25 percent and 11 percent, respectively ($p<0.01$). In females, spaying at <6 mo. and at 6-11mo. was associated with risks of 18 percent and 11 percent, respectively ($p<0.01$, when combined).

Cancers: lymphoma (LSA), mast cell tumor (MCT), hemangiosarcoma (HSA), and osteosarcoma (OSA). The occurrence of one or more of the cancers followed in intact males was a high 15 percent and neutering males at <6 mo. and at 6-11 mo. was associated with increased risks of 19 and 16 percent, respectively (both $p<0.01$). Leaving females intact resulted in a 5 percent occurrence in cancers, but spaying females at <6 mo. and at 6-11 mo., was associated with increases in cancers to 11 and 17 percent respectively ($p<0.05$, when combined). Spaying females at 1 year and 2-8 years was associated with an increased risk of 14 percent ($p<0.01$, when combined)

Mammary Cancer (MC), Pyometra (PYO) and Urinary Incontinence (UI) in Females. The occurrence of MC in intact females was 1 percent and for those spayed at 2-8 years, 4 percent. For females left intact, 4 percent were reported with PYO. No cases of UI were reported in females spayed at any age.

Bottom line. The suggested guideline for males, based on the increased risks of joint disorders and cancers, is delaying neutering until beyond a year of age. The suggested guideline for females, based on the increased occurrence of cancers at all spaying ages, is leaving the female intact or spaying after one year and remaining vigilant for the cancers.

Joint disorders. For ages 1 through 11 years and for each neuter period. Bold values indicate significance over the intact group. The dagger (†) indicates significance over the intact group when the early groups (< 6 mo. and 6-11 mo.) are combined.

	HD	CCL	ED	At Least One
Male < 6 months	15/97 (15.46)	8/111 (7.21)	5/106 (4.72)	**24/96 (25)** †
Male 6 – 11 months	5/106 (4.72)	4/117 (3.42)	4/110 (3.64)	**11/101 (10.89)** †
Male 1 year	1/44 (2.27)	0/49 (0)	0/46 (0)	1/42 (2.38)
Male 2 – 8 years	4/68 (5.88)	3/72 (4.17)	0/72 (0)	5/67 (7.46)
Male Intact	10/308 (3.25)	1/316 (0.32)	5/311 (1.61)	14/304 (4.61)
Female < 6 months	11/115 (9.57)	11/126 (8.73)	0/121 (0)	**20/113 (17.7)**†
Female 6 – 11 months	2/95 (2.11)	6/95 (6.32)	3/96 (3.13)	10/94 (10.64) †
Female 1 year	0/33 (0)	0/35 (0)	1/33 (3.03)	1/31 (3.23)
Female 2 – 8 years	4/100 (4)	3/103 (2.91)	0/102 (0)	7/99 (7.07)
Female Intact	6/188 (3.19)	0/190 (0)	2/189 (1.06)	8/189 (4.23)

Cancers. For ages 1 through 11 years and for each neuter period. Bold values indicate significance over the intact group. The dagger (†) indicates significance over the intact group when the early groups (< 6 mo. and 6-11 mo.) are combined.

	LSA	MCT	HSA	OSA	At Least One
Male < 6 months	12/109 (11.01)	3/111 (2.7)	6/112 (5.36)	1/111 (0.9)	**21/108 (19.44)**†
Male 6 – 11 months	9/117 (7.69)	4/117 (3.42)	4/117 (3.42)	1/118 (0.85)	**18/116 (15.52)** †
Male 1 year	0/49 (0)	1/48 (2.08)	1/46 (2.17)	3/49 (6.12)	4/46 (8.7)
Male 2 – 8 years	2/70 (2.86)	3/73 (4.11)	1/72 (1.39)	0/72 (0)	6/69 (8.7)
Male Intact	17/316 (5.38)	12/318 (3.77)	17/310 (5.48)	5/316 (1.58)	47/307 (15.31)
Female < 6 months	6/123 (4.88)	6/128 (4.69)	2/127 (1.57)	0/127 (0)	14/121 (11.57)†
Female 6 – 11 months	8/95 (8.42)	0/95 (0)	4/95 (4.21)	4/96 (4.17)	**16/93 (17.2)**†
Female 1 year	2/35 (5.71)	1/35 (2.86)	1/35 (2.86)	1/35 (2.86)	**5/35 (14.29)**
Female 2 – 8 years	2/97 (2.06)	8/103 (7.77)	2/97 (2.06)	1/102 (0.98)	13/93 (13.98)
Female Intact	5/190 (2.63)	1/190 (0.53)	3/190 (1.58)	0/190 (0)	9/190 (4.74)

Great Dane

The study population was 90 intact males, 103 neutered males, 69 intact females, and 91 spayed females for a total sample of 353 cases.

Joint disorders: hip dysplasia (HD), cranial cruciate ligament rupture (CCL), and elbow dysplasia (ED). Both males and females have a low level of joint disorders with those left intact having a 1 to 2 percent rate, respectively. For both males and females, there was no evident increase in this measure with neutering.

Cancers: lymphoma (LSA), mast cell tumor (MCT), hemangiosarcoma (HSA), and osteosarcoma (OSA). The occurrence of one or more of the cancers followed in intact males was 6 percent and for intact females, 3 percent. There was no significant increase in this measure of cancers with neutering in either sex.

Mammary Cancer (MC), Pyometra (PYO) and Urinary Incontinence (UI) in Females. In intact females, MC was diagnosed in just 2 percent and PYO, in 6 percent. In early-spayed females, no UI was reported.

Bottom line. Lacking a noticeable occurrence of increased joint disorders or cancers with neutering males or females, those wishing to neuter should decide on the appropriate age. However, given the large body size, and physiology of late musculoskeletal development, neutering well beyond year 1 should be considered.

Joint disorders. For ages 1 through 11 years and for each neuter period.

	HD	CCL	ED	At Least One
Male < 6 months	0/11 (0)	0/12 (0)	0/12 (0)	0/11 (0)
Male 6 – 11 months	0/39 (0)	0/39 (0)	1/40 (2.5)	1/38 (2.63)
Male 1 year	0/29 (0)	0/29 (0)	0/29 (0)	0/29 (0)
Male 2 – 8 years	0/20 (0)	0/22 (0)	0/22 (0)	0/20 (0)
Male Intact	1/90 (1.11)	0/89 (0)	1/89 (1.12)	2/88 (2.27)
Female < 6 months	1/19 (5.26)	0/19 (0)	0/19 (0)	1/19 (5.26)
Female 6 – 11 months	0/16 (0)	1/16 (6.25)	0/16 (0)	1/16 (6.25)
Female 1 year	0/27 (0)	1/29 (3.45)	0/29 (0)	1/27 (3.7)
Female 2 – 8 years	0/26 (0)	0/27 (0)	0/27 (0)	0/26 (0)
Female Intact	1/69 (1.45)	0/69 (0)	1/69 (1.45)	1/69 (1.45)

Cancers. For ages 1 through 11 years and for each neuter period.

	LSA	MCT	HSA	OSA	At Least One
Male < 6 months	0/12 (0)	0/12 (0)	0/12 (0)	0/11 (0)	0/11 (0)
Male 6 – 11 months	0/38 (0)	0/40 (0)	0/40 (0)	0/39 (0)	0/37 (0)
Male 1 year	1/29 (3.45)	1/29 (3.45)	1/28 (3.57)	0/28 (0)	3/28 (10.71)
Male 2 – 8 years	0/21 (0)	0/22 (0)	0/21 (0)	1/21 (4.76)	1/20 (5)
Male Intact	2/86 (2.33)	1/89 (1.12)	1/89 (1.12)	2/89 (2.25)	5/84 (5.95)
Female < 6 months	1/19 (5.26)	0/19 (0)	0/19 (0)	0/17 (0)	1/17 (5.88)
Female 6 – 11 months	0/16 (0)	0/16 (0)	0/15 (0)	0/16 (0)	0/15 (0)
Female 1 year	0/29 (0)	0/28 (0)	0/29 (0)	0/29 (0)	0/28 (0)
Female 2 – 8 years	0/27 (0)	0/27 (0)	0/27 (0)	1/27 (3.7)	1/27 (3.7)
Female Intact	0/69 (0)	0/69 (0)	0/69 (0)	2/69 (2.9)	2/69 (2.9)

Irish Wolfhound

The study population was 30 intact males, 19 neutered males, 21 intact females, and 16 spayed females for a total of 86 cases. Even with the small number of cases, this breed was chosen for analyses because of the large body size; challenging the Great Dane for height.

Joint disorders: hip dysplasia (HD), cranial cruciate ligament rupture (CCL), and elbow dysplasia (ED). In this sample, 7 percent of intact males and none of the intact females had a joint disorder. No joint disorders were seen in neutered males or females.

Cancers: lymphoma (LSA), mast cell tumor (MCT), hemangiosarcoma (HSA), and osteosarcoma (OSA). With the intact males and females, the incidences of one or more cancers were 8 percent and 21 percent, respectively. With neutering males at 1 year, there was an increase in cancer occurrence to 25 percent (p<0.05). With the high level of cancers in the intact females, there was no evident increase in cancers in spayed females.

Mammary Cancer (MC), Pyometra (PYO) and Urinary Incontinence (UI) in Females. There was no occurrence of MC in intact females or those spayed late. For females left intact, 5 percent were reported with PYO. UI was not reported in any of spayed or intact females.

Bottom line. With males, and the occurrence of cancers at 1-2 years, the suggested guideline of neutering is beyond 2 years. Lacking a noticeable occurrence of increased joint disorders or cancers with neutering females, those wishing to neuter should decide on the appropriate age. However, given the large body size, and physiology of late musculoskeletal development, neutering females well beyond year 1 should be considered.

.

Joint disorders. For ages 1 through 11 years and for each neuter period.

	HD	CCL	ED	At Least One
Male < 6 months	0/2 (0)	0/3 (0)	0/3 (0)	0/2 (0)
Male 6 – 11 months	0/2 (0)	0/2 (0)	0/2 (0)	0/2 (0)
Male 1 year	0/7 (0)	0/8 (0)	0/8 (0)	0/7 (0)
Male 2 – 8 years	0/6 (0)	0/6 (0)	0/6 (0)	0/6 (0)
Male Intact	1/31 (3.23)	0/30 (0)	1/28 (3.57)	2/29 (6.9)
Female < 6 months	0/1 (0)	0/1 (0)	0/1 (0)	0/1 (0)
Female 6 – 11 months	0/4 (0)	0/4 (0)	0/4 (0)	0/4 (0)
Female 1 year	0/3 (0)	0/3 (0)	0/3 (0)	0/3 (0)
Female 2 – 8 years	0/7 (0)	0/8 (0)	0/7 (0)	0/7 (0)
Female Intact	0/20 (0)	0/21 (0)	0/21 (0)	0/20 (0)

Cancers. For ages 1 through 11 years and for each neuter period. Bold values indicate significance over the intact group.

	LSA	MCT	HSA	OSA	At Least One
Male < 6 months	0/3 (0)	0/3 (0)	0/3 (0)	0/3 (0)	0/3 (0)
Male 6 – 11 months	1/1 (100)	0/2 (0)	0/2 (0)	0/1 (0)	1/1 (100)
Male 1 year	1/8 (12.5)	0/8 (0)	0/8 (0)	1/8 (12.5)	**2/8 (25)**
Male 2 – 8 years	0/6 (0)	0/6 (0)	0/5 (0)	1/6 (16.67)	1/6 (16.67)
Male Intact	0/29 (0)	0/30 (0)	0/29 (0)	2/28 (7.14)	2/26 (7.69)
Female < 6 months	0/1 (0)	0/1 (0)	0/1 (0)	0/1 (0)	0/1 (0)
Female 6 – 11 months	0/3 (0)	0/4 (0)	0/3 (0)	1/4 (25)	1/3 (33.33)
Female 1 year	0/2 (0)	0/3 (0)	0/2 (0)	0/2 (0)	0/1 (0)
Female 2 – 8 years	1/8 (12.5)	0/8 (0)	0/8 (0)	0/8 (0)	1/8 (12.5)
Female Intact	0/20 (0)	1/21 (4.76)	0/21 (0)	3/20 (15)	4/19 (21.05)

Jack Russell Terrier

The study population was 92 intact males, 87 neutered males, 84 intact females, and 113 spayed females for a total sample of 376 cases.

Joint disorders: hip dysplasia (HD), cranial cruciate ligament rupture (CCL), and elbow dysplasia (ED). Like other small dogs, joint disorders were rare; none of the intact males, and just 2 percent of intact females had one or more joint disorders. Neutering was not associated with any increase in this measure in either sex.

Cancers: lymphoma (LSA), mast cell tumor (MCT), hemangiosarcoma (HSA), and osteosarcoma (OSA). In intact males, 3 percent and in intact females none had one or more of the cancers followed. There was no evident increase in cancer occurrence in either sex with neutering at any age.

Mammary Cancer (MC), Pyometra (PYO) and Urinary Incontinence (UI) in Females. In females left intact, MC was seen in 1 percent, as was PYO. In those spayed at 2-8 years, MC was diagnosed in 3 percent. UI was not diagnosed in any females.

Bottom line. Lacking a noticeable occurrence of increased joint disorders or cancers with neutering males or females, those wishing to neuter should decide on the appropriate age.

Joint disorders. For ages 1 through 11 years and for each neuter period.

	HD	CCL	ED	At Least One
Male < 6 months	0/14 (0)	0/13 (0)	0/14 (0)	0/13 (0)
Male 6 – 11 months	0/28 (0)	0/27 (0)	0/28 (0)	0/27 (0)
Male 1 year	0/12 (0)	0/12 (0)	0/12 (0)	0/12 (0)
Male 2 – 8 years	0/32 (0)	1/32 (3.13)	0/32 (0)	1/32 (3.13)
Male Intact	0/92 (0)	0/91 (0)	0/92 (0)	0/91 (0)
Female < 6 months	0/17 (0)	0/17 (0)	0/18 (0)	0/16 (0)
Female 6 – 11 months	0/43 (0)	1/43 (2.33)	0/43 (0)	1/43 (2.33)
Female 1 year	0/16 (0)	0/16 (0)	0/16 (0)	0/16 (0)
Female 2 – 8 years	0/34 (0)	0/34 (0)	0/34 (0)	0/34 (0)
Female Intact	1/84 (1.19)	1/84 (1.19)	0/84 (0)	2/84 (2.38)

Cancers. For ages 1 through 11 years and for each neuter period.

	LSA	MCT	HSA	OSA	At Least One
Male < 6 months	0/14 (0)	1/14 (7.14)	0/14 (0)	0/14 (0)	1/14 (7.14)
Male 6 – 11 months	2/28 (7.14)	0/27 (0)	0/27 (0)	0/28 (0)	2/26 (7.69)
Male 1 year	0/12 (0)	0/12 (0)	1/12 (8.33)	0/12 (0)	1/12 (8.33)
Male 2 – 8 years	1/32 (3.13)	1/31 (3.23)	0/32 (0)	0/32 (0)	2/31 (6.45)
Male Intact	0/92 (0)	3/93 (3.23)	1/92 (1.09)	0/92 (0)	3/93 (3.23)
Female < 6 months	0/17 (0)	0/18 (0)	0/18 (0)	0/18 (0)	0/17 (0)
Female 6 – 11 months	0/41 (0)	1/43 (2.33)	1/43 (2.33)	0/43 (0)	2/41 (4.88)
Female 1 year	0/16 (0)	0/16 (0)	0/15 (0)	0/16 (0)	0/15 (0)
Female 2 – 8 years	0/33 (0)	2/34 (5.88)	0/34 (0)	0/34 (0)	2/33 (6.06)
Female Intact	0/83 (0)	0/84 (0)	0/84 (0)	0/84 (0)	0/83 (0)

Labrador Retriever

The study population was 714 intact males, 381 neutered males, 400 intact females, and 438 spayed females for a total of 1,933 cases.

Joint disorders: hip dysplasia (HD), cranial cruciate ligament rupture (CCL), and elbow dysplasia (ED). One or more joint disorders were reported in 6 percent of both intact males and intact females. This measure was significantly increased to 13 percent for males neutered before 6 mo. (p<0.01). In females spayed at <6 mo. and 6-11 mo., the risk of a joint disorder was 11-12 percent for each period (p<0.01, spay periods combined).

Cancers: lymphoma (LSA), mast cell tumor (MCT), hemangiosarcoma (HSA), and osteosarcoma (OSA). The occurrences of cancers were 8 percent and 6 percent for intact males and females, respectively, with no evident increased risk in a cancer with neutering.

Mammary Cancer (MC), Pyometra (PYO) and Urinary Incontinence (UI) in Females. The occurrence of MC in intact females was 1 percent and for those spayed at 2-8 years, 2 percent. For females left intact, 2 percent were reported with PYO. UI was reported at a low rate (2-3 percent) in females spayed at various ages though 1 year.

Bottom line. For males, given increase in joint disorders at the < 6 mo. neutering, the suggested guideline is neutering beyond 6 months of age. For females, given the increased risks of joint disorders with spaying through 1 year of age, the suggested guideline is delaying spaying until beyond a year of age.

Joint disorders. For ages 1 through 11 years and for each neuter period. Bold values indicate significance over the intact group. The asterisk (*) indicates when there was significance using the Wilcoxon test, but no significance using the log-rank test. The dagger (†) indicates significance over the intact group when the early groups (< 6 mo. and 6-11 mo.) are combined.

	HD	CCL	ED	At Least One
Male < 6 months	0/54 (0)	4/58 (6.9)	2/49 (4.08)	**6/48 (12.5)** †
Male 6 – 11 months	1/93 (1.08)	3/100 (3)	1/92 (1.09)	5/88 (5.68)
Male 1 year	2/65 (3.08)	1/69 (1.45)	0/65 (0)	3/63 (4.76)
Male 2 – 8 years	1/128 (0.78)	1/128 (0.78)	2/127 (1.57)	4/124 (3.23)
Male Intact	15/703 (2.13)	17/701 (2.43)	8/702 (1.14)	40/684 (5.85)
Female < 6 months	3/56 (5.36)	3/59 (5.08)	1/57 (1.75)	**6/53 (11.32)*** †
Female 6 – 11 months	4/120 (3.33)	9/122 (7.38)	0/125 (0)	13/113 (11.5) †
Female 1 year	4/69 (5.8)	0/71 (0)	0/72 (0)	4/68 (5.88)
Female 2 – 8 years	1/154 (0.65)	4/153 (2.61)	0/157 (0)	5/147 (3.4)
Female Intact	7/398 (1.76)	11/399 (2.76)	4/398 (1.01)	23/396 (5.81)

Cancers. For ages 1 through 11 years and for each neuter period.

	LSA	MCT	HSA	OSA	At Least One
Male < 6 months	0/58 (0)	2/59 (3.39)	0/59 (0)	0/59 (0)	2/58 (3.45)
Male 6 – 11 months	1/98 (1.02)	0/100 (0)	1/100 (1)	0/99 (0)	2/96 (2.08)
Male 1 year	1/69 (1.45)	2/69 (2.9)	2/67 (2.99)	0/68 (0)	5/67 (7.46)
Male 2 – 8 years	2/128 (1.56)	7/125 (5.6)	1/128 (0.78)	2/129 (1.55)	12/122 (9.84)
Male Intact	10/696 (1.44)	22/711 (3.09)	10/697 (1.43)	6/706 (0.85)	46/678 (6.78)
Female < 6 months	1/60 (1.67)	0/60 (0)	0/60 (0)	0/60 (0)	1/60 (1.67)
Female 6 – 11 months	1/123 (0.81)	5/126 (3.97)	1/127 (0.79)	1/127 (0.79)	7/123 (5.69)
Female 1 year	0/70 (0)	1/71 (1.41)	0/70 (0)	0/71 (0)	1/67 (1.49)
Female 2 – 8 years	2/155 (1.29)	8/152 (5.26)	1/157 (0.64)	1/157 (0.64)	12/148 (8.11)
Female Intact	7/395 (1.77)	10/399 (2.51)	2/397 (0.5)	3/395 (0.76)	22/389 (5.66)

Maltese

The study population was 49 intact males, 72 neutered males, 65 intact females, and 86 spayed females for a total sample of 272 cases. The Maltese and Chihuahua vie for the smallest breeds and the Great Dane and Irish Wolfhound for the largest. Interestingly, while the two extremes vary greatly in size, they seem to share the same low predisposition to joint disorders.

Joint disorders: hip dysplasia (HD), cranial cruciate ligament rupture (CCL), and elbow dysplasia (ED). Quite noteworthy, in both sexes there was no occurrence of any joint disorders in either those left intact or neutered.

Cancers: lymphoma (LSA), mast cell tumor (MCT), hemangiosarcoma (HSA), and osteosarcoma (OSA). Virtually the same picture emerges with cancers as with joints. Only one of 64 intact females was diagnosed with a cancer.

Mammary Cancer (MC), Pyometra (PYO) and Urinary Incontinence (UI) in Females. There was no occurrence of MC in the intact females and only one occurrence among the 19 females spayed at 2-8 years. PYO was seen in none of the intact females. UI did not occur in any of the females.

Bottom line. Lacking a noticeable occurrence of increased joint disorders or cancers with neutering males or females, those wishing to neuter should decide on the appropriate age.

Joint disorders. For ages 1 through 11 years and for each neuter period.

	HD	CCL	ED	At Least One
Male < 6 months	0/13 (0)	0/13 (0)	0/12 (0)	0/12 (0)
Male 6 – 11 months	0/27 (0)	0/27 (0)	0/27 (0)	0/27 (0)
Male 1 year	0/10 (0)	0/10 (0)	0/10 (0)	0/10 (0)
Male 2 – 8 years	0/20 (0)	0/20 (0)	0/20 (0)	0/20 (0)
Male Intact	0/49 (0)	0/49 (0)	0/49 (0)	0/49 (0)
Female < 6 months	0/14 (0)	0/14 (0)	0/14 (0)	0/14 (0)
Female 6 – 11 months	0/40 (0)	0/40 (0)	0/40 (0)	0/40 (0)
Female 1 year	0/12 (0)	0/12 (0)	0/12 (0)	0/12 (0)
Female 2 – 8 years	0/19 (0)	0/19 (0)	0/19 (0)	0/19 (0)
Female Intact	0/65 (0)	0/65 (0)	0/65 (0)	0/65 (0)

Cancers. For ages 1 through 11 years and for each neuter period.

	LSA	MCT	HSA	OSA	At Least One
Male < 6 months	0/11 (0)	0/13 (0)	0/13 (0)	0/13 (0)	0/11 (0)
Male 6 – 11 months	0/26 (0)	0/27 (0)	0/27 (0)	0/27 (0)	0/26 (0)
Male 1 year	0/10 (0)	0/10 (0)	0/10 (0)	0/10 (0)	0/10 (0)
Male 2 – 8 years	0/19 (0)	0/20 (0)	0/20 (0)	0/20 (0)	0/19 (0)
Male Intact	0/47 (0)	0/49 (0)	0/49 (0)	0/49 (0)	0/47 (0)
Female < 6 months	0/14 (0)	0/14 (0)	0/14 (0)	0/14 (0)	0/14 (0)
Female 6 – 11 months	0/39 (0)	0/40 (0)	0/40 (0)	0/40 (0)	0/39 (0)
Female 1 year	0/12 (0)	0/12 (0)	0/12 (0)	0/12 (0)	0/12 (0)
Female 2 – 8 years	0/18 (0)	0/19 (0)	0/19 (0)	0/19 (0)	0/18 (0)
Female Intact	1/64 (1.56)	0/65 (0)	0/65 (0)	0/65 (0)	1/64 (1.56)

Miniature Schnauzer
The study population was 47 intact males, 63 neutered males, 25 intact females, and 95 spayed females for a total sample of 230 cases.

Joint disorders: hip dysplasia (HD), cranial cruciate ligament rupture (CCL), and elbow dysplasia (ED). There was virtually no occurrence of any joint disorder in males or females, either left intact or neutered.

Cancers: lymphoma (LSA), mast cell tumor (MCT), hemangiosarcoma (HSA), and osteosarcoma (OSA). The incidence of cancers in intact males was 4 percent and in females, zero percent. There was no indication of cancer occurrence related to neutering in either sex.

Mammary Cancer (MC), Pyometra (PYO) and Urinary Incontinence (UI) in Females. There was no occurrence of MC in any of the females left intact or spayed. PYO was diagnosed in 4 percent of intact females. None of the females were diagnosed with UI.

Bottom line. Lacking a noticeable occurrence of increased joint disorders or cancers with neutering males or females, those wishing to neuter should decide on the appropriate age.

Joint disorders. For ages 1 through 11 years and for each neuter period.

	HD	CCL	ED	At Least One
Male < 6 months	0/10 (0)	0/10 (0)	0/10 (0)	0/10 (0)
Male 6 – 11 months	0/19 (0)	0/19 (0)	0/19 (0)	0/19 (0)
Male 1 year	0/13 (0)	0/13 (0)	0/13 (0)	0/13 (0)
Male 2 – 8 years	0/17 (0)	0/17 (0)	0/17 (0)	0/17 (0)
Male Intact	0/47 (0)	0/47 (0)	0/47 (0)	0/47 (0)
Female < 6 months	0/18 (0)	0/18 (0)	0/18 (0)	0/18 (0)
Female 6 – 11 months	0/42 (0)	1/42 (2.38)	0/42 (0)	1/42 (2.38)
Female 1 year	0/11 (0)	0/11 (0)	0/11 (0)	0/11 (0)
Female 2 – 8 years	0/23 (0)	0/23 (0)	0/23 (0)	0/23 (0)
Female Intact	0/25 (0)	0/25 (0)	0/25 (0)	0/25 (0)

Cancers. For ages 1 through 11 years and for each neuter period.

	LSA	MCT	HSA	OSA	At Least One
Male < 6 months	0/10 (0)	0/10 (0)	0/10 (0)	0/10 (0)	0/10 (0)
Male 6 – 11 months	0/18 (0)	0/18 (0)	1/19 (5.26)	0/19 (0)	1/17 (5.88)
Male 1 year	0/13 (0)	0/13 (0)	0/13 (0)	0/13 (0)	0/13 (0)
Male 2 – 8 years	0/17 (0)	0/17 (0)	0/17 (0)	0/17 (0)	0/17 (0)
Male Intact	2/48 (4.17)	0/47 (0)	0/47 (0)	0/47 (0)	2/48 (4.17)
Female < 6 months	0/17 (0)	0/17 (0)	0/18 (0)	0/18 (0)	0/17 (0)
Female 6 – 11 months	0/37 (0)	0/41 (0)	0/41 (0)	0/42 (0)	0/37 (0)
Female 1 year	0/10 (0)	0/11 (0)	0/11 (0)	0/11 (0)	0/10 (0)
Female 2 – 8 years	0/22 (0)	1/22 (4.55)	0/23 (0)	0/23 (0)	1/22 (4.55)
Female Intact	0/24 (0)	0/25 (0)	0/25 (0)	0/25 (0)	0/24 (0)

Pomeranian

The study population was 84 intact males, 69 neutered males, 65 intact females, and 104 spayed females for a total sample of 322 cases.

Joint disorders: hip dysplasia (HD), cranial cruciate ligament rupture (CCL), and elbow dysplasia (ED). As with other dogs of small body size, there was no occurrence of any joint disorder in either those left intact or neutered.

Cancers: lymphoma (LSA), mast cell tumor (MCT), hemangiosarcoma (HSA), and osteosarcoma (OSA). The occurrence of cancers was zero for males and females left intact, and there was no indication of increased cancer risk related to neutering in either sex.

Mammary Cancer (MC), Pyometra (PYO) and Urinary Incontinence (UI) in Females. There was one case of MC in those left intact, and 7 percent of these had PYO. None of the females was diagnosed with UI.

Bottom line. Lacking a noticeable occurrence of increased joint disorders or cancers with neutering males or females, those wishing to neuter should decide on the appropriate age.

Joint disorders. For ages 1 through 11 years and for each neuter period.

	HD	CCL	ED	At Least One
Male < 6 months	0/7 (0)	0/7 (0)	0/7 (0)	0/7 (0)
Male 6 – 11 months	0/24 (0)	0/24 (0)	0/24 (0)	0/24 (0)
Male 1 year	0/14 (0)	0/14 (0)	0/14 (0)	0/14 (0)
Male 2 – 8 years	0/23 (0)	0/22 (0)	0/23 (0)	0/22 (0)
Male Intact	0/84 (0)	0/84 (0)	0/84 (0)	0/84 (0)
Female < 6 months	0/10 (0)	0/10 (0)	0/10 (0)	0/10 (0)
Female 6 – 11 months	0/30 (0)	0/31 (0)	0/31 (0)	0/30 (0)
Female 1 year	0/18 (0)	0/18 (0)	0/17 (0)	0/17 (0)
Female 2 – 8 years	0/39 (0)	0/38 (0)	0/39 (0)	0/38 (0)
Female Intact	0/64 (0)	0/65 (0)	0/65 (0)	0/64 (0)

Cancers. For ages 1 through 11 years and for each neuter period.

	LSA	MCT	HSA	OSA	At Least One
Male < 6 months	0/7 (0)	0/7 (0)	0/7 (0)	0/7 (0)	0/7 (0)
Male 6 – 11 months	0/24 (0)	1/24 (4.17)	0/24 (0)	0/24 (0)	1/24 (4.17)
Male 1 year	0/14 (0)	0/14 (0)	0/14 (0)	0/14 (0)	0/14 (0)
Male 2 – 8 years	0/23 (0)	0/23 (0)	0/23 (0)	0/23 (0)	0/23 (0)
Male Intact	0/84 (0)	0/84 (0)	0/83 (0)	0/84 (0)	0/83 (0)
Female < 6 months	0/10 (0)	0/10 (0)	0/10 (0)	0/10 (0)	0/10 (0)
Female 6 – 11 months	0/31 (0)	0/30 (0)	0/31 (0)	0/31 (0)	0/30 (0)
Female 1 year	0/18 (0)	0/18 (0)	0/18 (0)	0/18 (0)	0/18 (0)
Female 2 – 8 years	0/37 (0)	0/39 (0)	0/39 (0)	0/39 (0)	0/37 (0)
Female Intact	0/64 (0)	0/65 (0)	0/65 (0)	0/65 (0)	0/64 (0)

Poodle, Toy

The study population was 49 intact males, 53 neutered males, 58 intact females, and 78 spayed females for a total sample of 238 cases. While the AKC registers all the Poodle varieties as the same breed, the three main varieties are dealt with separately here because of differences in size.

Joint disorders: hip dysplasia (HD), cranial cruciate ligament rupture (CCL), and elbow dysplasia (ED). In intact males, 4 percent had one or more joint disorders, and in males neutered at 6-11 mo., just a single case was diagnosed with a joint disorder. In intact and spayed females there was no occurrence of a joint disorder.

Cancers: lymphoma (LSA), mast cell tumor (MCT), hemangiosarcoma (HSA), and osteosarcoma (OSA). There was a 2 percent occurrence of cancers in intact males and a non-significant, single case occurrence of a cancer among those neutered at 6-11 mo. There was no occurrence of a cancer in intact or spayed females.

Mammary Cancer (MC), Pyometra (PYO) and Urinary Incontinence (UI) in Females. The only occurrence of MC was in 1 of the 57 intact females. There were no cases of PYO in intact females and no occurrence of UI in spayed females

Bottom line. Lacking a noticeable occurrence of increased joint disorders or cancers with neutering males or females, those wishing to neuter should decide on the appropriate age.

Joint disorders. For ages 1 through 11 years and for each neuter period.

	HD	CCL	ED	At Least One
Male < 6 months	0/14 (0)	0/14 (0)	0/14 (0)	0/14 (0)
Male 6 – 11 months	0/14 (0)	1/14 (7.14)	0/14 (0)	1/14 (7.14)
Male 1 year	0/10 (0)	0/10 (0)	0/10 (0)	0/10 (0)
Male 2 – 8 years	0/10 (0)	0/11 (0)	0/11 (0)	0/10 (0)
Male Intact	1/49 (2.04)	1/49 (2.04)	0/49 (0)	2/49 (4.08)
Female < 6 months	0/14 (0)	0/14 (0)	0/14 (0)	0/14 (0)
Female 6 – 11 months	0/29 (0)	0/29 (0)	0/29 (0)	0/29 (0)
Female 1 year	0/12 (0)	0/11 (0)	0/12 (0)	0/11 (0)
Female 2 – 8 years	0/18 (0)	0/18 (0)	0/18 (0)	0/18 (0)
Female Intact	0/58 (0)	0/58 (0)	0/58 (0)	0/58 (0)

Cancers. For ages 1 through 11 years and for each neuter period.

	LSA	MCT	HSA	OSA	At Least One
Male < 6 months	0/14 (0)	0/14 (0)	0/14 (0)	0/14 (0)	0/14 (0)
Male 6 – 11 months	0/14 (0)	1/14 (7.14)	0/14 (0)	0/14 (0)	1/14 (7.14)
Male 1 year	0/9 (0)	0/10 (0)	0/10 (0)	0/10 (0)	0/9 (0)
Male 2 – 8 years	0/11 (0)	0/11 (0)	0/11 (0)	0/11 (0)	0/11 (0)
Male Intact	1/50 (2)	0/49 (0)	0/49 (0)	0/49 (0)	1/50 (2)
Female < 6 months	0/14 (0)	0/14 (0)	0/14 (0)	0/14 (0)	0/14 (0)
Female 6 – 11 months	0/28 (0)	0/29 (0)	0/29 (0)	0/29 (0)	0/28 (0)
Female 1 year	0/12 (0)	0/12 (0)	0/12 (0)	0/12 (0)	0/12 (0)
Female 2 – 8 years	0/18 (0)	0/18 (0)	0/18 (0)	0/18 (0)	0/18 (0)
Female Intact	0/58 (0)	0/58 (0)	0/58 (0)	0/58 (0)	0/58 (0)

Poodle, Miniature

The study population was 41 intact males, 60 neutered males, 30 intact females, and 69 spayed females for a total sample of 199 cases. The AKC registers the Toy, Miniature, and Standard Poodle varieties as all being the same breed. However, because of differences in size, the varieties of Poodles are dealt with separately here.

Joint disorders: hip dysplasia (HD), cranial cruciate ligament rupture (CCL), and elbow dysplasia (ED). There was no occurrence of joint disorders in intact males or intact females. In males neutered at 6-11 mo., there was a significant 9 percent occurrence of joint disorders (p<0.01), reflecting CCL diagnoses. In spayed females, there was no occurrence of a joint disorder.

Cancers: lymphoma (LSA), mast cell tumor (MCT), hemangiosarcoma (HSA), and osteosarcoma (OSA). In intact male and females, there were 5 and zero percent occurrences of cancers, respectively. There was no indication of an increased cancer occurrence related to neutering in either sex.

Mammary Cancer (MC), Pyometra (PYO) and Urinary Incontinence (UI) in Females. The only occurrence of MC was one female spayed at 2-8 years. Of intact females, 6 percent developed PYO. Just one female spayed at <6 mo. developed UI.

Bottom line. The suggested guideline for males, based on the significant occurrence of a joint disorder with neutering at 6-11 months, is delaying neutering until a year of age. Lacking a noticeable occurrence of increased joint disorders or cancers with neutering females, those wishing to neuter should decide on the appropriate age.

Joint disorders. For ages 1 through 11 years and for each neuter period. Bold values indicate significance over the intact group. The dagger (†) indicates significance over the intact group when the early groups (< 6 mo. and 6-11 mo.) are combined.

	HD	CCL	ED	At Least One
Male < 6 months	0/11 (0)	0/11 (0)	0/11 (0)	0/11 (0) †
Male 6 – 11 months	0/23 (0)	2/24 (8.33)	0/24 (0)	**2/23 (8.7)** †
Male 1 year	0/6 (0)	0/6 (0)	0/6 (0)	0/6 (0)
Male 2 – 8 years	0/14 (0)	0/14 (0)	0/14 (0)	0/14 (0)
Male Intact	0/41 (0)	0/41 (0)	0/41 (0)	0/41 (0)
Female < 6 months	0/14 (0)	0/14 (0)	0/14 (0)	0/14 (0)
Female 6 – 11 months	0/23 (0)	0/23 (0)	0/23 (0)	0/23 (0)
Female 1 year	0/12 (0)	0/12 (0)	0/12 (0)	0/12 (0)
Female 2 – 8 years	0/17 (0)	0/17 (0)	0/17 (0)	0/17 (0)
Female Intact	0/30 (0)	0/30 (0)	0/30 (0)	0/30 (0)

Cancers. For ages 1 through 11 years and for each neuter period.

	LSA	MCT	HSA	OSA	At Least One
Male < 6 months	0/11 (0)	0/11 (0)	0/11 (0)	0/11 (0)	0/11 (0)
Male 6 – 11 months	0/24 (0)	0/24 (0)	0/24 (0)	0/24 (0)	0/24 (0)
Male 1 year	0/6 (0)	0/6 (0)	0/6 (0)	0/6 (0)	0/6 (0)
Male 2 – 8 years	0/13 (0)	0/14 (0)	0/14 (0)	0/14 (0)	0/13 (0)
Male Intact	1/42 (2.38)	1/41 (2.44)	0/41 (0)	0/41 (0)	2/42 (4.76)
Female < 6 months	0/14 (0)	0/14 (0)	0/14 (0)	0/14 (0)	0/14 (0)
Female 6 – 11 months	0/22 (0)	0/23 (0)	0/22 (0)	0/23 (0)	0/21 (0)
Female 1 year	0/12 (0)	0/12 (0)	0/12 (0)	0/12 (0)	0/12 (0)
Female 2 – 8 years	1/16 (6.25)	0/17 (0)	0/17 (0)	0/16 (0)	1/15 (6.67)
Female Intact	0/29 (0)	0/30 (0)	0/30 (0)	0/30 (0)	0/29 (0)

Poodle, Standard

The study population was 47 intact males, 88 neutered males, 53 intact females, and 87 spayed females for a total sample of 275 cases. The AKC registers the Toy and Miniature, along with the Standard Poodle, as all being Poodles. However, because of differences in size, the varieties of Poodles are dealt with separately here.

Joint disorders: hip dysplasia (HD), cranial cruciate ligament rupture (CCL), and elbow dysplasia (ED). There was a 2 percent occurrence of joint disorders in both intact males and females. In males neutered at <6 mo., there was a non-significant increase to 8 percent, and in spayed females there was no occurrence of joint disorders.

Cancers: lymphoma (LSA), mast cell tumor (MCT), hemangiosarcoma (HSA), and osteosarcoma (OSA). The occurrences of cancers in intact males and females were 4 and 2 percent, respectively. In males neutered at 1 year of age, the occurrence rose to a significant 27 percent ($p<0.01$), all due to the increased risk of LSA. In females, there was no significant increase in cancers with spaying.

Mammary Cancer (MC), Pyometra (PYO) and Urinary Incontinence (UI) in Females. There was a 4 percent occurrence of MC, and a 2 percent occurrence of PYO in the females left intact. Just one female spayed beyond 2 years later developed UI.

Bottom line. The suggested guideline for males, based on the occurrence of a cancer with neutering at 1 year, is to delay neutering until 2 years of age. Lacking a noticeable occurrence of increased joint disorders or cancers with neutering females, those wishing to neuter should decide on the appropriate age.

Joint disorders. For ages 1 through 11 years and for each neuter period.

	HD	CCL	ED	At Least One
Male < 6 months	2/24 (8.33)	0/25 (0)	0/24 (0)	2/24 (8.33)
Male 6 – 11 months	0/38 (0)	1/38 (2.63)	0/38 (0)	1/38 (2.63)
Male 1 year	0/16 (0)	0/16 (0)	0/16 (0)	0/16 (0)
Male 2 – 8 years	0/8 (0)	0/8 (0)	0/8 (0)	0/8 (0)
Male Intact	1/47 (2.13)	0/47 (0)	0/47 (0)	1/47 (2.13)
Female < 6 months	0/17 (0)	0/18 (0)	0/18 (0)	0/17 (0)
Female 6 – 11 months	0/30 (0)	0/30 (0)	0/31 (0)	0/29 (0)
Female 1 year	0/10 (0)	0/10 (0)	0/10 (0)	0/10 (0)
Female 2 – 8 years	0/28 (0)	0/28 (0)	0/28 (0)	0/28 (0)
Female Intact	1/53 (1.89)	0/52 (0)	0/53 (0)	1/52 (1.92)

Cancers. For ages 1 through 11 years and for each neuter period. Bold values indicate significance over the intact group.

	LSA	MCT	HSA	OSA	At Least One
Male < 6 months	2/25 (8)	0/25 (0)	0/25 (0)	0/25 (0)	2/25 (8)
Male 6 – 11 months	0/37 (0)	0/38 (0)	1/36 (2.78)	0/37 (0)	1/36 (2.78)
Male 1 year	4/16 (25)	0/16 (0)	0/15 (0)	0/16 (0)	**4/15 (26.67)**
Male 2 – 8 years	0/8 (0)	0/7 (0)	0/8 (0)	0/8 (0)	0/7 (0)
Male Intact	1/47 (2.13)	1/47 (2.13)	0/47 (0)	0/47 (0)	2/47 (4.26)
Female < 6 months	0/17 (0)	0/18 (0)	0/18 (0)	0/18 (0)	0/17 (0)
Female 6 – 11 months	1/31 (3.23)	0/31 (0)	0/31 (0)	0/31 (0)	1/31 (3.23)
Female 1 year	1/9 (11.11)	0/10 (0)	0/10 (0)	0/10 (0)	1/9 (11.11)
Female 2 – 8 years	0/26 (0)	0/27 (0)	1/28 (3.57)	0/28 (0)	1/25 (4)
Female Intact	1/53 (1.89)	0/53 (0)	0/53 (0)	0/53 (0)	1/53 (1.89)

Pug

The study population was 96 intact males, 106 neutered males, 63 intact females, and 117 spayed females for a total sample of 382 cases.

Joint disorders: hip dysplasia (HD), cranial cruciate ligament rupture (CCL), and elbow dysplasia (ED). In intact males and females, the occurrences of joint disorders were zero and 2 percent, respectively. In neutered males, there was no occurrence of a joint disorder, and in spayed females there was an insignificant occurrence to 5 percent having a joint disorder for those spayed at <6 mo. and 6-11 mo.

Cancers: lymphoma (LSA), mast cell tumor (MCT), hemangiosarcoma (HSA), and osteosarcoma (OSA). The level of occurrence of one or more cancers in intact males was 6 percent and in intact females, 8 percent. Neutering males and females did not lead to any evident increase in risk of a cancer.

Mammary Cancer (MC), Pyometra (PYO) and Urinary Incontinence (UI) in Females. There were no cases of MC in females left intact or spayed at any time. PYO was diagnosed in 5 percent of intact females. None of the females was diagnosed with UI.

Bottom line. Lacking a noticeable occurrence of increased joint disorders or cancers with neutering males or females, those wishing to neuter should decide on the appropriate age.

Joint disorders. For ages 1 through 11 years and for each neuter period.

	HD	CCL	ED	At Least One
Male < 6 months	0/16 (0)	0/16 (0)	0/16 (0)	0/16 (0)
Male 6 – 11 months	0/40 (0)	0/40 (0)	0/40 (0)	0/40 (0)
Male 1 year	0/22 (0)	0/22 (0)	0/22 (0)	0/22 (0)
Male 2 – 8 years	0/23 (0)	0/23 (0)	0/23 (0)	0/23 (0)
Male Intact	0/95 (0)	0/96 (0)	0/96 (0)	0/95 (0)
Female < 6 months	1/20 (5)	0/21 (0)	0/21 (0)	1/20 (5)
Female 6 – 11 months	1/42 (2.38)	1/44 (2.27)	0/45 (0)	2/41 (4.88)
Female 1 year	0/13 (0)	0/13 (0)	0/13 (0)	0/13 (0)
Female 2 – 8 years	1/36 (2.78)	0/37 (0)	0/37 (0)	1/36 (2.78)
Female Intact	1/63 (1.59)	0/63 (0)	0/63 (0)	1/63 (1.59)

Cancers. For ages 1 through 11 years and for each neuter period.

	LSA	MCT	HSA	OSA	At Least One
Male < 6 months	0/16 (0)	0/16 (0)	0/16 (0)	0/16 (0)	0/16 (0)
Male 6 – 11 months	1/40 (2.5)	2/39 (5.13)	0/39 (0)	0/40 (0)	3/38 (7.89)
Male 1 year	0/21 (0)	0/22 (0)	0/22 (0)	0/22 (0)	0/21 (0)
Male 2 – 8 years	0/23 (0)	1/20 (5)	0/23 (0)	0/23 (0)	1/20 (5)
Male Intact	2/93 (2.15)	4/98 (4.08)	0/95 (0)	0/95 (0)	6/94 (6.38)
Female < 6 months	1/21 (4.76)	2/21 (9.52)	0/21 (0)	0/21 (0)	3/21 (14.29)
Female 6 – 11 months	0/44 (0)	5/44 (11.36)	0/45 (0)	0/45 (0)	5/43 (11.63)
Female 1 year	0/13 (0)	0/13 (0)	0/13 (0)	0/13 (0)	0/13 (0)
Female 2 – 8 years	0/36 (0)	0/36 (0)	0/37 (0)	0/37 (0)	0/35 (0)
Female Intact	0/61 (0)	5/64 (7.81)	0/63 (0)	0/63 (0)	5/62 (8.06)

Rottweiler
The study population was 315 intact males, 152 neutered males, 143 intact females, and 239 spayed females for a total sample of 854 cases.

Joint disorders: hip dysplasia (HD), cranial cruciate ligament rupture (CCL), and elbow dysplasia (ED). Joint disorders are a major concern in this breed with 8 percent of intact males and 16 percent of intact females having one or more joint disorders. In males, neutering at <6 mo. and at 6-11 mo. resulted in diagnoses for 10 percent and 22 percent, respectively, with the combined occurrences being significantly above the intact occurrences ($p<0.05$). In females, spaying at <6 mo. was associated with a significant 43 percent occurrence ($p<0.05$). In females, the main joint disorder associated with spaying was CCL.

Cancers: lymphoma (LSA), mast cell tumor (MCT), hemangiosarcoma (HSA), and osteosarcoma (OSA). The cancers followed occurred in the intact males and females at 16 and 11 percent, respectively. This relatively high occurrence in intact males and females was not significantly increased by neutering at any age.

Mammary Cancer (MC), Pyometra (PYO) and Urinary Incontinence (UI) in Females. Of females left intact or spayed at 2-8 years, 8 and 5 percent were diagnosed with MC, respectively. In intact females, 12 percent were diagnosed with PYO. Also in intact females, 1 percent had UI, and in females spayed at <6 mo. and 6-11 mo., 4 and 6 percent respectively had UI.

Bottom line. For males, given the risk of joint disorders for those neutered at 6-11 months or earlier, the suggested guideline is neutering beyond a year of age. For females, the suggested guideline is spaying beyond 6 months of age, which avoids the increase in joint disorders for those spayed at < 6 months.

Joint disorders. For ages 1 through 11 years and for each neuter period. Bold values indicate significance over the intact group. The asterisk (*) indicates when there was significance using the Wilcoxon test, but no significance using the log-rank test. The dagger (†) indicates significance over the intact group when the early groups (< 6 mo. and 6-11 mo.) are combined.

	HD	CCL	ED	At Least One
Male < 6 months	0/12 (0)	1/10 (10)	0/12 (0)	1/10 (10)†
Male 6 – 11 months	2/26 (7.69)	2/27 (7.41)	2/27 (7.41)	**5/23 (21.74)*** †
Male 1 year	1/40 (2.5)	1/45 (2.22)	0/43 (0)	2/37 (5.41)
Male 2 – 8 years	1/47 (2.13)	2/52 (3.85)	0/52 (0)	2/44 (4.55)
Male Intact	8/308 (2.6)	12/307 (3.91)	6/306 (1.96)	23/297 (7.74)
Female < 6 months	0/24 (0)	8/28 (28.57)	1/28 (3.57)	**9/21 (42.86)** †
Female 6 – 11 months	1/52 (1.92)	9/53 (16.98)	1/54 (1.85)	9/47 (19.15)†
Female 1 year	0/44 (0)	1/45 (2.22)	2/43 (4.65)	3/41 (7.32)
Female 2 – 8 years	0/85 (0)	3/85 (3.53)	0/91 (0)	2/75 (2.67)
Female Intact	8/143 (5.59)	11/143 (7.69)	6/140 (4.29)	24/147 (16.33)

Cancers. For ages 1 through 11 years and for each neuter period.

	LSA	MCT	HSA	OSA	At Least One
Male < 6 months	0/13 (0)	0/13 (0)	0/13 (0)	0/13 (0)	0/13 (0)
Male 6 – 11 months	1/28 (3.57)	0/29 (0)	0/28 (0)	3/30 (10)	4/28 (14.29)
Male 1 year	0/44 (0)	0/46 (0)	1/45 (2.22)	0/44 (0)	1/42 (2.38)
Male 2 – 8 years	3/53 (5.66)	1/53 (1.89)	0/50 (0)	1/52 (1.92)	4/48 (8.33)
Male Intact	19/307 (6.19)	3/315 (0.95)	3/309 (0.97)	21/300 (7)	46/292 (15.75)
Female < 6 months	0/31 (0)	0/31 (0)	0/32 (0)	1/31 (3.23)	1/30 (3.33)
Female 6 – 11 months	1/53 (1.89)	0/56 (0)	0/57 (0)	5/57 (8.77)	6/51 (11.76)
Female 1 year	2/47 (4.26)	0/47 (0)	1/47 (2.13)	2/47 (4.26)	5/47 (10.64)
Female 2 – 8 years	4/87 (4.6)	0/89 (0)	1/88 (1.14)	2/90 (2.22)	7/76 (9.21)
Female Intact	11/140 (7.86)	2/145 (1.38)	0/143 (0)	3/140 (2.14)	16/140 (11.43)

Saint Bernard

The study population was 26 intact males, 27 neutered males, 18 intact females, and 23 spayed females for a total sample of 94 cases.

Joint disorders: hip dysplasia (HD), cranial cruciate ligament rupture (CCL), and elbow dysplasia (ED). In intact males and females, the occurrences of one or more joint disorders were 8 percent and 6 percent, respectively. Neutering males at <6 mo., resulted in two occurrences (40 percent, non-significant). In females spayed at <6 mo., joint disorders increased to a significant 100 percent ($p<0.01$).

Cancers: lymphoma (LSA), mast cell tumor (MCT), hemangiosarcoma (HSA), and osteosarcoma (OSA). The cancers followed occurred in the intact males and females at 4 and 11 percent, respectively. With neutering males and females there was no significant increase in this measure.

Mammary Cancer (MC), Pyometra (PYO) and Urinary Incontinence (UI) in Females. There were no occurrences of MC in either the intact or spayed females. In intact females, PYO was diagnosed in 15 percent. There was no occurrence of UI in spayed females.

Bottom line. Lacking a noticeable occurrence of increased joint disorders or cancers with neutering males those wishing to neuter should decide on the appropriate age. The suggested guideline for females given and increase in joint disorders with spaying at < 6 mo., is neutering beyond 6 months. However, given the large body size, neutering well beyond 1 year should be considered in both sexes.

Joint disorders. For ages 1 through 11 years and for each neuter period. Bold values indicate significance over the intact group. The dagger (†) indicates significance over the intact group when the early groups (< 6 mo. and 6-11 mo.) are combined.

	HD	CCL	ED	At Least One
Male < 6 months	1/5 (20)	1/5 (20)	0/5 (0)	2/5 (40)
Male 6 – 11 months	0/6 (0)	1/7 (14.29)	1/7 (14.29)	1/7 (14.29)
Male 1 year	0/5 (0)	0/6 (0)	0/6 (0)	0/5 (0)
Male 2 – 8 years	0/8 (0)	0/7 (0)	0/8 (0)	0/7 (0)
Male Intact	1/24 (4.17)	1/27 (3.7)	0/26 (0)	2/25 (8)
Female < 6 months	2/4 (50)	1/4 (25)	2/4 (50)	**4/4 (100)** †
Female 6 – 11 months	0/4 (0)	0/5 (0)	0/4 (0)	0/3 (0) †
Female 1 year	0/5 (0)	0/5 (0)	0/5 (0)	0/5 (0)
Female 2 – 8 years	0/8 (0)	0/8 (0)	0/8 (0)	0/8 (0)
Female Intact	1/18 (5.56)	0/18 (0)	0/18 (0)	1/18 (5.56)

Cancers. For ages 1 through 11 years and for each neuter period.

	LSA	MCT	HSA	OSA	At Least One
Male < 6 months	1/6 (16.67)	0/6 (0)	0/6 (0)	1/6 (16.67)	1/6 (16.67)
Male 6 – 11 months	0/7 (0)	0/7 (0)	0/7 (0)	0/7 (0)	0/7 (0)
Male 1 year	0/4 (0)	0/6 (0)	0/6 (0)	0/6 (0)	0/4 (0)
Male 2 – 8 years	0/7 (0)	0/7 (0)	0/8 (0)	0/8 (0)	0/6 (0)
Male Intact	0/25 (0)	0/26 (0)	0/26 (0)	1/26 (3.85)	1/25 (4)
Female < 6 months	0/4 (0)	0/4 (0)	0/4 (0)	0/4 (0)	0/4 (0)
Female 6 – 11 months	0/4 (0)	0/5 (0)	0/5 (0)	1/4 (25)	1/3 (33.33)
Female 1 year	0/5 (0)	0/5 (0)	0/5 (0)	0/5 (0)	0/5 (0)
Female 2 – 8 years	0/8 (0)	0/8 (0)	0/7 (0)	1/8 (12.5)	1/7 (14.29)
Female Intact	0/18 (0)	0/18 (0)	0/18 (0)	2/18 (11.11)	2/18 (11.11)

Shetland Sheepdog
The study population was 31 intact males, 30 neutered males, 20 intact females, and 52 spayed females for a total sample of 133 cases.

Joint disorders: hip dysplasia (HD), cranial cruciate ligament rupture (CCL), and elbow dysplasia (ED). There were no joint disorders in intact males and was just one case in the intact females. In neutered males, the only joint disorder was in one of the males neutered at <6 mo. In females, there was no joint disorder associated with spaying.

Cancers: lymphoma (LSA), mast cell tumor (MCT), hemangiosarcoma (HSA), and osteosarcoma (OSA). The occurrence of cancers in intact males was 6 percent. At the 6-11 mo. interval, 14 percent of males were diagnosed with one or more cancers (not significant). None of the intact females was diagnosed with a cancer, but 25 percent of females spayed at <6 mo. were diagnosed with one or more cancer (not significant).

Mammary Cancer (MC), Pyometra (PYO) and Urinary Incontinence (UI) in Females. There was no occurrence of MC in intact or spayed females and was a 14 percent occurrence of PYO in intact females. Spaying at 6-11 mo. resulted in a 6 percent occurrence of UI, and at 1 year a 33 percent occurrence of UI.

Bottom line. Lacking a noticeable occurrence of increased joint disorders or cancers with neutering males, those wishing to neuter should decide on the appropriate age. However, to avoid the high level of UI occurrence in females, one could consider spaying females at, or beyond, 2 years.

Joint disorders. For ages 1 through 11 years and for each neuter period.

	HD	CCL	ED	At Least One
Male < 6 months	1/5 (20)	0/5 (0)	0/5 (0)	1/5 (20)
Male 6 – 11 months	0/14 (0)	0/14 (0)	0/13 (0)	0/13 (0)
Male 1 year	0/2 (0)	0/2 (0)	0/2 (0)	0/2 (0)
Male 2 – 8 years	0/4 (0)	0/4 (0)	0/4 (0)	0/4 (0)
Male Intact	0/31 (0)	0/31 (0)	0/31 (0)	0/31 (0)
Female < 6 months	0/7 (0)	0/7 (0)	0/8 (0)	0/6 (0)
Female 6 – 11 months	0/21 (0)	0/21 (0)	0/21 (0)	0/21 (0)
Female 1 year	0/7 (0)	0/7 (0)	0/7 (0)	0/7 (0)
Female 2 – 8 years	0/15 (0)	0/15 (0)	0/15 (0)	0/15 (0)
Female Intact	0/19 (0)	0/20 (0)	1/20 (5)	1/19 (5.26)

Cancers. For ages 1 through 11 years and for each neuter period.

	LSA	MCT	HSA	OSA	At Least One
Male < 6 months	0/5 (0)	0/5 (0)	0/5 (0)	0/5 (0)	0/5 (0)
Male 6 – 11 months	1/14 (7.14)	1/14 (7.14)	0/14 (0)	0/14 (0)	2/14 (14.29)
Male 1 year	0/2 (0)	0/2 (0)	0/2 (0)	0/2 (0)	0/2 (0)
Male 2 – 8 years	0/3 (0)	0/4 (0)	0/4 (0)	0/4 (0)	0/3 (0)
Male Intact	1/32 (3.13)	1/32 (3.13)	0/31 (0)	0/31 (0)	2/33 (6.06)
Female < 6 months	1/8 (12.5)	0/8 (0)	1/8 (12.5)	0/8 (0)	2/8 (25)
Female 6 – 11 months	1/21 (4.76)	0/20 (0)	0/21 (0)	0/21 (0)	1/20 (5)
Female 1 year	0/7 (0)	0/7 (0)	0/7 (0)	0/7 (0)	0/7 (0)
Female 2 – 8 years	0/15 (0)	0/15 (0)	0/15 (0)	0/15 (0)	0/15 (0)
Female Intact	0/20 (0)	0/20 (0)	0/20 (0)	0/20 (0)	0/20 (0)

Shih Tzu

The study population was 104 intact males, 112 neutered males, 77 intact females, and 139 spayed females for a total sample of 432 cases.

Joint disorders: hip dysplasia (HD), cranial cruciate ligament rupture (CCL), and elbow dysplasia (ED). There was no occurrence of a joint disorder in either intact or neutered males and females, revealing virtually no vulnerability in this regard.

Cancers: lymphoma (LSA), mast cell tumor (MCT), hemangiosarcoma (HSA), and osteosarcoma (OSA). There was no occurrence of the cancers followed in intact and neutered males or in intact females. However, in females spayed at 6-11 mo. the occurrence of cancers was 7 percent (not significant), but at 1 year this measure reached a significant 18 percent (p<0.01).

Mammary Cancer (MC), Pyometra (PYO) and Urinary Incontinence (UI) in Females. MC occurred in 3 percent of intact females, with no occurrence in spayed females. PYO occurred in 5 percent of intact females. UI was not reported in any females.

Bottom line. Lacking a noticeable occurrence of increased joint disorders or cancers with neutering males, those wishing to neuter should decide on the appropriate age. The picture is different for spaying females where the risk of the cancers followed reached a significant 18 percentage at year 1. The suggested guideline for females is to delay spaying until the female is 2 years of age. Another possibility is spaying a female a month or two before 6 months to avoid the increased risk of cancers of spaying later.

Joint disorders. For ages 1 through 11 years and for each neuter period.

	HD	CCL	ED	At Least One
Male < 6 months	0/19 (0)	0/19 (0)	0/18 (0)	0/18 (0)
Male 6 – 11 months	0/45 (0)	0/45 (0)	0/45 (0)	0/45 (0)
Male 1 year	0/20 (0)	0/20 (0)	0/20 (0)	0/20 (0)
Male 2 – 8 years	0/25 (0)	0/25 (0)	0/25 (0)	0/25 (0)
Male Intact	0/103 (0)	0/104 (0)	0/104 (0)	0/103 (0)
Female < 6 months	0/22 (0)	0/22 (0)	0/22 (0)	0/22 (0)
Female 6 – 11 months	0/47 (0)	0/47 (0)	0/47 (0)	0/47 (0)
Female 1 year	0/17 (0)	0/16 (0)	0/17 (0)	0/16 (0)
Female 2 – 8 years	0/47 (0)	0/47 (0)	0/47 (0)	0/47 (0)
Female Intact	0/77 (0)	0/77 (0)	0/77 (0)	0/77 (0)

Cancers. For ages 1 through 11 years and for each neuter period. Bold values indicate significance over the intact group.

	LSA	MCT	HSA	OSA	At Least One
Male < 6 months	0/19 (0)	0/19 (0)	0/19 (0)	0/19 (0)	0/19 (0)
Male 6 – 11 months	0/44 (0)	0/45 (0)	0/45 (0)	0/44 (0)	0/44 (0)
Male 1 year	0/20 (0)	0/20 (0)	0/20 (0)	0/20 (0)	0/20 (0)
Male 2 – 8 years	0/25 (0)	0/25 (0)	0/25 (0)	0/25 (0)	0/25 (0)
Male Intact	0/104 (0)	0/104 (0)	0/104 (0)	0/104 (0)	0/104 (0)
Female < 6 months	0/22 (0)	0/22 (0)	0/22 (0)	0/22 (0)	0/22 (0)
Female 6 – 11 months	2/46 (4.35)	1/47 (2.13)	0/46 (0)	0/47 (0)	3/46 (6.52)
Female 1 year	2/17 (11.76)	1/17 (5.88)	0/17 (0)	0/17 (0)	**3/17 (17.65)**
Female 2 – 8 years	0/45 (0)	0/47 (0)	0/47 (0)	0/47 (0)	0/45 (0)
Female Intact	0/76 (0)	0/77 (0)	0/77 (0)	0/77 (0)	0/76 (0)

West Highland White Terrier

The study population was 35 intact males, 33 neutered males, 28 intact females, and 46 spayed females for a total sample of 142 cases.

Joint disorders: hip dysplasia (HD), cranial cruciate ligament rupture (CCL), and elbow dysplasia (ED). Just one intact male had a joint disorder, and other than this, no joint disorders were reported in intact females or in neutered males or females.

Cancers: lymphoma (LSA), mast cell tumor (MCT), hemangiosarcoma (HSA), and osteosarcoma (OSA). None of the intact males or females had any of the cancers followed. The only occurrence of the cancers followed was one case of LSA in a female spayed at 6-11 mo.

Mammary Cancer (MC), Pyometra (PYO) and Urinary Incontinence (UI) in Females. There were no occurrences of MC in either intact or neutered females, and was a 7 percent occurrence of PYO in intact females. The occurrences of UI were 14 percent for females spayed at <6 mo. and 6 percent with spaying at 6-11 mo.

Bottom line. Lacking a noticeable occurrence of increased joint disorders or cancers with neutering males or females, those wishing to neuter should decide on the appropriate age. However, for females, one could consider delaying spaying until a year of age to avoid the risk of UI.

Joint disorders. For ages 1 through 11 years and for each neuter period.

	HD	CCL	ED	At Least One
Male < 6 months	0/5 (0)	0/5 (0)	0/5 (0)	0/5 (0)
Male 6 – 11 months	0/17 (0)	0/17 (0)	0/17 (0)	0/17 (0)
Male 1 year	0/4 (0)	0/4 (0)	0/4 (0)	0/4 (0)
Male 2 – 8 years	0/5 (0)	0/5 (0)	0/5 (0)	0/5 (0)
Male Intact	0/35 (0)	1/35 (2.86)	0/35 (0)	1/35 (2.86)
Female < 6 months	0/7 (0)	0/7 (0)	0/7 (0)	0/7 (0)
Female 6 – 11 months	0/16 (0)	0/15 (0)	0/16 (0)	0/15 (0)
Female 1 year	0/5 (0)	0/5 (0)	0/5 (0)	0/5 (0)
Female 2 – 8 years	0/14 (0)	0/14 (0)	0/14 (0)	0/14 (0)
Female Intact	0/28 (0)	0/28 (0)	0/28 (0)	0/28 (0)

Cancers. For ages 1 through 11 years and for each neuter period.

	LSA	MCT	HSA	OSA	At Least One
Male < 6 months	0/5 (0)	0/5 (0)	0/5 (0)	0/5 (0)	0/5 (0)
Male 6 – 11 months	0/17 (0)	0/17 (0)	0/17 (0)	0/17 (0)	0/17 (0)
Male 1 year	0/4 (0)	0/4 (0)	0/4 (0)	0/4 (0)	0/4 (0)
Male 2 – 8 years	0/5 (0)	0/5 (0)	0/5 (0)	0/5 (0)	0/5 (0)
Male Intact	0/34 (0)	0/35 (0)	0/35 (0)	0/35 (0)	0/34 (0)
Female < 6 months	0/7 (0)	0/7 (0)	0/7 (0)	0/7 (0)	0/7 (0)
Female 6 – 11 months	1/16 (6.25)	0/16 (0)	0/16 (0)	0/16 (0)	1/16 (6.25)
Female 1 year	0/5 (0)	0/5 (0)	0/5 (0)	0/5 (0)	0/5 (0)
Female 2 – 8 years	0/14 (0)	0/14 (0)	0/14 (0)	0/14 (0)	0/14 (0)
Female Intact	0/28 (0)	0/28 (0)	0/28 (0)	0/28 (0)	0/28 (0)

Yorkshire Terrier

The study population was 134 intact males, 178 neutered males, 144 intact females, and 229 spayed females for a total sample of 685 cases.

Joint disorders: hip dysplasia (HD), cranial cruciate ligament rupture (CCL), and elbow dysplasia (ED). There was no joint disorder reported in intact or neutered males. In intact females, this measure occurred in 1 percent. In females spayed at all the intervals from <6 mo. through 2-8 years, 2-4 percent had a joint disorder (CCL), none of which was significantly above that of intact females.

Cancers: lymphoma (LSA), mast cell tumor (MCT), hemangiosarcoma (HSA), and osteosarcoma (OSA). In intact males and intact females, 1 percent were reported with at least one of the cancers followed. In both males and females neutered at 6-11 mo. and at 2-8 years, 1 to 3 percent had a cancer (mostly due to LSA). None of the cancer occurrences was noteworthy.

Mammary Cancer (MC), Pyometra (PYO) and Urinary Incontinence (UI) in Females. In intact females, the occurrence of MC was 1 percent as was the occurrence with spaying at 2-8 years. PYO was reported in 7 percent of intact females. No UI was reported in any of the intact or spayed females.

Bottom line. Lacking a noticeable occurrence of increased joint disorders or cancers with neutering males or females, those wishing to neuter should decide on the appropriate age.

Joint disorders. For ages 1 through 11 years and for each neuter period.

	HD	CCL	ED	At Least One
Male < 6 months	0/39 (0)	0/38 (0)	0/39 (0)	0/38 (0)
Male 6 – 11 months	0/69 (0)	0/69 (0)	0/69 (0)	0/69 (0)
Male 1 year	0/33 (0)	0/33 (0)	0/33 (0)	0/33 (0)
Male 2 – 8 years	0/34 (0)	0/33 (0)	0/34 (0)	0/33 (0)
Male Intact	0/133 (0)	0/133 (0)	0/134 (0)	0/132 (0)
Female < 6 months	0/25 (0)	1/26 (3.85)	0/26 (0)	1/25 (4)
Female 6 – 11 months	0/90 (0)	3/89 (3.37)	0/90 (0)	3/89 (3.37)
Female 1 year	0/40 (0)	1/38 (2.63)	0/40 (0)	1/38 (2.63)
Female 2 – 8 years	0/69 (0)	1/66 (1.52)	0/69 (0)	1/66 (1.52)
Female Intact	0/143 (0)	2/143 (1.4)	0/144 (0)	2/142 (1.41)

Cancers. For ages 1 through 11 years and for each neuter period.

	LSA	MCT	HSA	OSA	At Least One
Male < 6 months	0/37 (0)	0/39 (0)	0/38 (0)	0/39 (0)	0/36 (0)
Male 6 – 11 months	1/65 (1.54)	0/69 (0)	0/69 (0)	0/68 (0)	1/64 (1.56)
Male 1 year	0/33 (0)	0/33 (0)	0/33 (0)	0/33 (0)	0/33 (0)
Male 2 – 8 years	1/34 (2.94)	0/34 (0)	0/34 (0)	0/34 (0)	1/34 (2.94)
Male Intact	1/132 (0.76)	0/132 (0)	0/134 (0)	0/134 (0)	1/131 (0.76)
Female < 6 months	0/25 (0)	0/26 (0)	0/26 (0)	0/26 (0)	0/25 (0)
Female 6 – 11 months	1/90 (1.11)	0/89 (0)	0/90 (0)	0/89 (0)	1/88 (1.14)
Female 1 year	0/38 (0)	0/40 (0)	0/40 (0)	0/40 (0)	0/38 (0)
Female 2 – 8 years	1/66 (1.52)	1/69 (1.45)	0/69 (0)	0/69 (0)	2/66 (3.03)
Female Intact	2/141 (1.42)	0/144 (0)	0/143 (0)	0/144 (0)	2/140 (1.43)
