# Supplementary material for: Assisting Decision-Making on Age of Neutering for 35 Breeds of Dogs: Associated Joint Disorders, Cancers, and Urinary Incontinence
Source: Front Vet Sci. 2020 Jul 7;7:388. doi: 10.3389/fvets.2020.00388 (PMC7359819; doi:10.3389/fvets.2020.00388)
Supplement: Supplementary file 2 [file Data_Sheet_2.pdf]

Appendix 2. Mean Age at Last Visit. For neutered males (MN), spayed females (FS), intact males (MI), and intact females (FI), the table lists the mean age at last visit within the study range by breed group. The number of neutered and intact females and males for each breed group is also included. The bottom of the table includes the calculation for the overall mean age at last visit within the study range for all dogs included in the study.

	**FS Mean**	**FS No.**	**FI Mean**	**FI No.**	**MN Mean**	**MN No.**	**MI Mean**	**MI No.**
Australian Cattle Dog	5.68	70	4.8	48	4.81	58	6.4	61
Australian Shepherd	6.04	136	4.96	76	5.9	135	6.38	93
Beagle	5.69	87	5.7	45	5.96	82	6.7	42
Bernese Mt. Dog	5.19	65	4.39	37	4.48	74	4.81	59
Border Collie	5.3	121	5.02	88	6.38	85	6.29	105
Boston Terrier	5.04	96	4.75	54	5.05	67	6.32	75
Boxer	5.47	210	4.09	128	5.31	203	5.74	220
Bulldog	5.36	114	3.41	90	4.38	156	4.15	198
Cavalier King Charles Spaniel	5.48	76	4.03	87	5.45	72	5.09	51
Chihuahua	4.83	289	4.25	298	4.65	189	4.85	261
Cocker Spaniel	6.81	127	5.18	61	6.46	112	7.11	71
Collie	6.97	37	5.28	24	6.52	26	6.38	29
Corgi	6.55	70	4.43	50	5.85	78	4.87	42
Dachshund	6.32	212	5.18	99	6.36	170	6.23	177
Doberman Pinscher	5.7	108	4.93	53	6	91	5.73	106
English Springer Spaniel	6.1	66	5.35	37	5.77	57	6.04	52
German Shepherd	5.64	298	4.94	173	5.43	272	5.6	514
Golden Retriever	5.9	374	4.51	190	5.82	365	6.72	318
Great Dane	4.49	91	3.98	69	3.99	103	4.22	90
Irish Wolfhound	4.21	16	5.04	21	4.64	19	5.16	30
Jack Russell Terrier	5.4	113	4.89	84	5.85	87	5.59	92
Labrador Retriever	6.05	438	4.95	400	5.77	381	6.07	714
Maltese	5.14	86	4.9	65	5.66	72	5.54	49
Miniature Schnauzer	6.3	96	5.29	25	5.72	63	6.24	47
Pomeranian	5.82	104	4.65	65	5.99	69	6	84
Poodle (Toy)	5.94	78	4.48	58	5.94	53	5.56	49
Poodle (Miniature)	5.53	69	4.8	30	5.8	60	6.19	41
Poodle (Standard)	6.09	87	3.7	53	5.59	88	5.56	47
Pug	5.93	118	4.59	63	5.31	106	5.65	96
Rottweiler	5.56	239	5.04	143	5.29	152	6.37	315
Saint Bernard	5.08	23	4.15	18	3.71	27	4.88	26
Shetland Sheepdog	6.22	52	6.32	20	6.54	30	6.42	31
Shih Tzu	6.08	139	4.94	77	5.1	112	6.08	104
West Highland White Terrier	6.51	46	5	28	6.05	33	6.69	35
Yorkshire Terrier	5.34	229	4.59	144	5.06	178	5.25	134
**OVERALL**	**5.7**	**4580**	**4.7**	**3001**	**5.5**	**3925**	**4.9**	**4458**
